# Modeling the inner part of the jet in M87: Confronting jet morphology with theory

**DOI:** 10.1126/sciadv.adn3544

**Published:** 2024-03-22

**Authors:** Hai Yang, Feng Yuan, Hui Li, Yosuke Mizuno, Fan Guo, Rusen Lu, Luis C. Ho, Xi Lin, Andrzej A. Zdziarski, Jieshuang Wang

**Affiliations:** ^1^ Shanghai Astronomical Observatory, Chinese Academy of Sciences; 80 Nandan Road, Shanghai 200030, China.; ^2^ University of Chinese Academy of Sciences; 19A Yuquan Road, Beijing 100049, China.; ^3^ Tsung-Dao Lee Institute, Shanghai Jiao Tong University, Shengrong Road 520, Shanghai 201210, China.; ^4^ Center for Astronomy and Astrophysics and Department of Physics, Fudan University, Shanghai 200438, China.; ^5^ Los Alamos National Laboratory, Los Alamos, NM 87545, USA.; ^6^ School of Physics and Astronomy, Shanghai Jiao Tong University, 800 Dongchuan Road, Shanghai 200240, China.; ^7^ Institut für Theoretische Physik, Goethe-Universität Frankfurt, Max-von-Laue-Str. 1, D-60438 Frankfurt am Main, Germany.; ^8^ Key Laboratory of Radio Astronomy and Technology, Chinese Academy of Sciences, A20 Datun Road, Chaoyang District, Beijing 100101, PR China.; ^9^ Max-Planck-Institut für Radioastronomie, Auf dem Hügel 69, D-53121 Bonn, Germany.; ^10^ Kavli Institute for Astronomy and Astrophysics, Peking University, Beijing 100871, China.; ^11^ Department of Astronomy, School of Physics, Peking University, Beijing 100871, China.; ^12^ Nicolaus Copernicus Astronomical Center, Polish Academy of Sciences, Bartycka 18, PL-00-716 Warszawa, Poland.; ^13^ Max-Planck-Institut für Kernphysik, Saupfercheckweg 1, D-69117 Heidelberg, Germany.

## Abstract

The formation of jets in black hole accretion systems is a long-standing problem. It has been proposed that a jet can be formed by extracting the rotation energy of the black hole (“BZ-jet”) or the accretion flow (“disk-jet”). While both models can produce collimated relativistic outflows, neither has successfully explained the observed jet morphology. By using general relativistic magnetohydrodynamic simulations and considering nonthermal electrons accelerated by magnetic reconnection that is likely driven by magnetic eruption in the underlying accretion flow, we obtain images by radiative transfer calculations and compared them to millimeter observations of the jet in M87. We find that the BZ-jet originating from a magnetically arrested disk around a high-spin black hole can well reproduce the jet morphology, including its width and limb-brightening feature.

## Teaser

The jet driven by extracting the rotation energy of the black hole can well explain the observed morphology of the jet in M87.

## INTRODUCTION

How a jet forms from a black hole accretion flow has been an important unsolved problem. The current consensus is that jet formation requires a combination of magnetic fields and rotation. The most influential model (BZ-jet) is that of Blandford-Znajek (*1*), in which the jet is formed by extracting the spin energy of the black hole via the magnetic field lines connected to its event horizon. The analytical predictions of this scenario have been confirmed by general relativistic magnetohydrodynamic (GRMHD) simulations that successfully produce relativistic collimated outflows (*2*–*5*). However, it has been unclear whether the BZ-jet can explain the observed morphology of the jet, including its elongated structure, width, and limb-brightening.

As an alternative to black hole spin, the jet can also be powered by extracting the rotation energy of the accretion flow by the magnetic centrifugal force or the gradient of the magnetic pressure (*6*, *7*). Such a disk-jet model has also received support from numerical simulations (*8*–*10*). Which of these two leading competing scenarios matches the jets observed in active galactic nuclei?

## RESULTS AND DISCUSSION

This work uses three-dimensional GRMHD simulations to reproduce the detailed structure of the jet in M87 to test its formation mechanism. We simulate a hot accretion flow around a black hole using the ATHENA++ code (*11*). The details of how we perform the simulations and the numerical convergence test are presented in the “Three-dimensional GRMHD numerical simulation of a hot accretion flow” and “Convergence of numerical resolution” sections in Materials and Methods, respectively. Because the black hole in M87 is likely rapidly rotating and the accretion flow is likely to be a magnetically arrested disk (MAD) instead of undergoing standard and normal evolution (SANE) (*12*, *13*), our fiducial model is a MAD around black holes with spin *a* = 0.98 (MAD98). For comparison, we also simulate a SANE around a black hole with *a* = 0.98 (SANE98) and two MADs around black holes with *a* = 0.5 (MAD05) and *a* = 0 (MAD00). The two-dimensional distributions of several physical quantities of MAD98 are shown in fig. S2. Specifically, the red line in each panel of fig. S2 denotes the boundary of the BZ-jet.

To calculate the radiation of the jet, we need to quantify the electron distribution, including both the thermal and nonthermal components. Because our MHD simulation is for a single fluid, we calculate the temperature of thermal electrons following the approach presented in (*14*), which is a function of the plasma β (the ratio between gas and magnetic pressure) and two other free parameters *R*_low_ and *R*_high_ (the “Determination of distributions of thermal and nonthermal electrons” section in Materials and Methods). A large fraction of radiation of the jet is believed to originate from nonthermal electrons, but the specific mechanism of electron acceleration has been a mystery. Here, we assume that the mechanism is magnetic reconnection. Numerical simulations have found that MADs are subject to magnetic flux eruptions that occur when a magnetic flux bundle containing strong vertical fields escapes from the black hole’s magnetosphere and propagates radially outward into the disk (*4*, *15*–*19*). These events must strongly perturb the vertical magnetic field lines and can in principle trigger magnetic reconnection in the jet. We have conducted several quantitative analyses and confirmed this scenario (in the “Physical origin of magnetic reconnection in the jet” section in Materials and Methods).

In light of the particle acceleration by magnetic reconnection (*20*–*24*), we assume that the accelerated electrons follow a power-law energy distribution. Details on how we determine the minimum Lorentz factor (γ_min_) and power-law index (*p*) of the electron energy distribution are given in the “Determination of distributions of thermal and nonthermal electrons” section in Materials and Methods. For our problem, one of the most important parameters is the amount of nonthermal electrons and their spatial distribution. In the present work, we adopt the description proposed by Petersen *et al.* (*25*), which assumes that acceleration rate is proportional to (**J**/*J*_0_)^2^, where** J** is the current density and *J*_0_ is a characteristic current density characterizing the property of the background plasma. The physical motivation of such a prescription is based on particle-in-cell (PIC) simulations of particle acceleration (*25*). On the other hand, we would like to emphasize that this is still a simplified prescription to particle acceleration by reconnection because it relies on volumetric current rather than the current in the reconnecting current sheets. Radiative cooling is also considered when we try to obtain the number density of nonthermal electrons (the “Determination of distributions of thermal and nonthermal electrons” section in Materials and Methods). This description distinguishes our model from others. For instance, in (*26*), the number density of nonthermal electrons is roughly a constant percentage of the number density of thermal electrons, with mainly the spectral index of nonthermal electrons changing from place to place, depending on the local values of parameters β and σ.

**Fig. 1.**
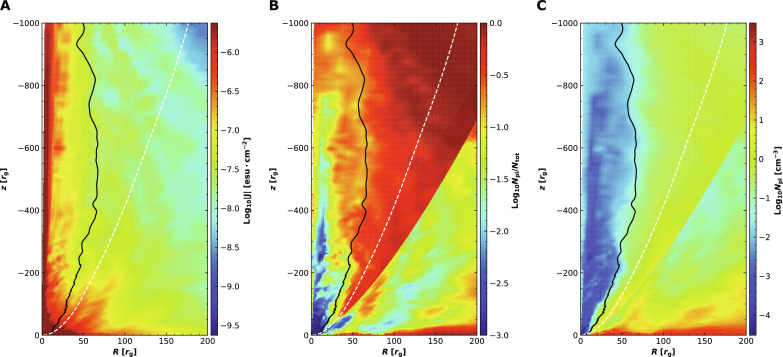
The **ϕ**-averaged two-dimensional spatial distribution of some quantities of MAD98 at a simulation time of *t* = 27,400. From left to right, the colors denote the logarithm of (**A**) the three-current density **J,** (**B**) the ratio of number density of nonthermal and total electrons *N*_pl_/*N*_tot_, and (**C**) the number density of nonthermal (power-law) electrons *N*_pl_. In each panel the black solid line denotes σ = 5, while the white dashed line denotes the ϕ-averaged boundary of the BZ-jet. In the middle panel, there is a sharp feature to the right of the white dashed line. This is due to the different scaling of *r_z_* in Eq. 6 adopted in our model. Its presence will not affect our results.

The three panels in Fig. 1 show the two-dimensional distribution of **J**, the ratio of the number density of nonthermal (power-law) electrons and total electrons *N*_pl_/*N*_tot_, and the number density of nonthermal electrons *N*_pl_. We can see that *N*_pl_/*N*_tot_ reaches its largest values in a layer around the boundary of the BZ-jet. Note that the largest value of *N*_pl_/*N*_tot_ is close to but smaller than one. We find that most of the jet radiation comes from the part of the above-mentioned layer inside of the BZ-jet, and the narrowness of the radiation layer is one of the reasons for the limb-brightening feature we will show later. The number density of nonthermal electrons is largest in the equatorial plane of the accretion flow because the gas density there is much larger than in the jet. This does not imply that the radio emission from the accretion flow will dominate the jet because the magnetic field in the accretion flow is weaker than in the jet (fig. S2).

**Fig. 2.**
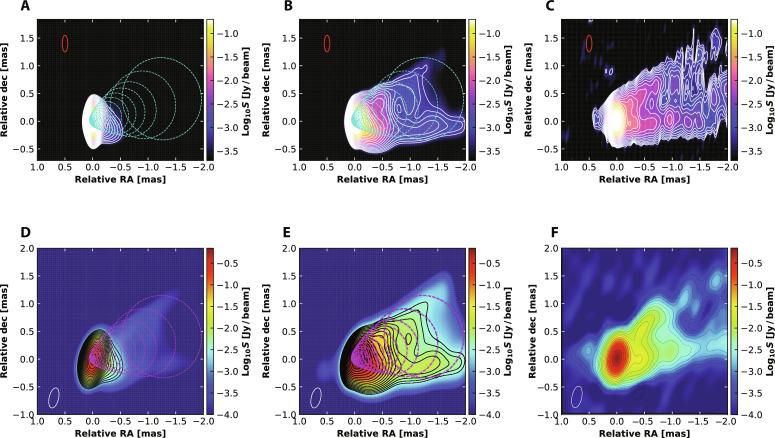
Comparison of images predicted by models and observations. (**A** to **C**) Images at 86 GHz. (**D** to **F**) Images at 43 GHz. The left, middle, and right panels are the images predicted by (A and D) the thermal-only model, (B and E) the fiducial current-density model, and (C and F) the observed images, respectively. The observational data are taken from (*64*) (86 GHz) and (*65*) (43 GHz). The model images are calculated by convolving the simulations with a beam of 0.3 × 0.1 mas (top) and 0.37 × 0.17 mas (bottom) at −13.3° to mimic the limited resolution of the telescopes used in the observations. The dynamical ranges of the predicted and observed images are the same. See the “Calculation of radiative transfer in the jet” section in Materials and Methods for details for calculating the images. The dotted circles denote the boundary of the BZ-jet.

**Fig. 3.**
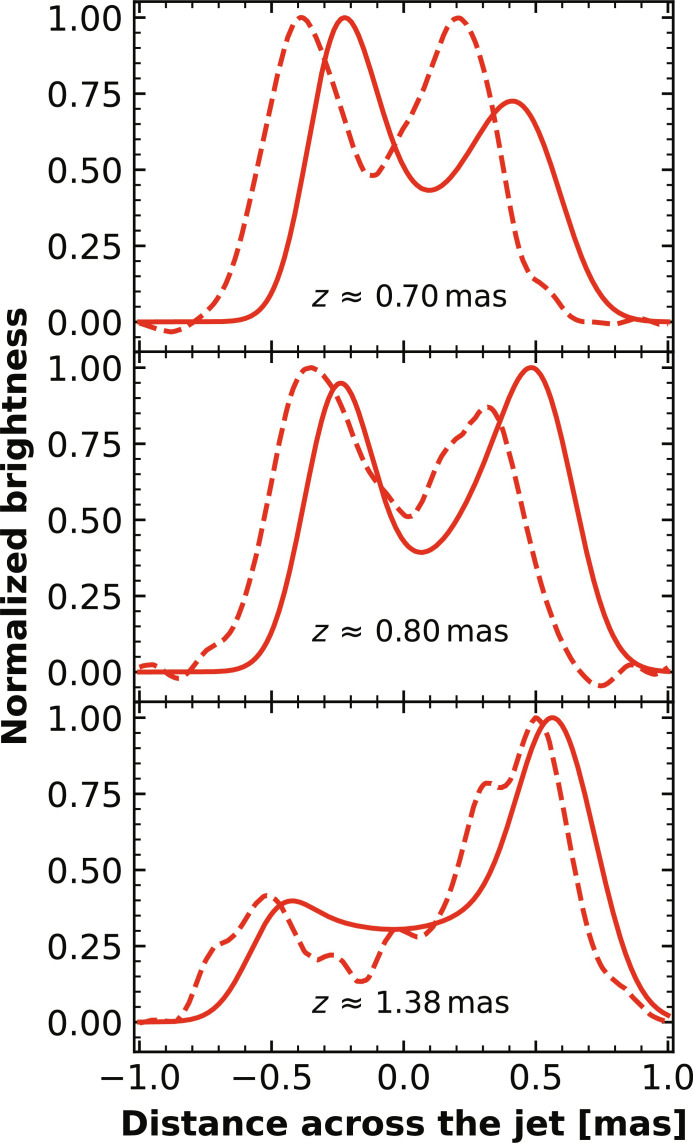
The limb-brightening predicted by the fiducial model (solid lines) and its comparison with observations (dashed lines) at three distances from the core. The observational data are based on Global mm-VLBI Array (GMVA) observations at 86 GHz (*64*). As the flux fluctuates with time for both the models and observations, we have normalized the predicted flux to the observed value.

We use the general relativistic radiative transfer code IPOLE (*27*) to calculate the predicted images of the jet (“Calculation of radiative transfer in the jet” section in Materials and Methods). We tested 50 snapshots from the simulation data of MAD98 and found that our results are systematically and substantially better than previous works, in the sense that the jet is longer, its width is more consistent with observations, and its limb brightening is much more evident. Figure 2 compares the representative model images at 86 and 43 GHz, demonstrating that only the “current density” model successfully reproduces the opening angle of the jet and the elongated structure up to distance ∼2 mas from the core, while a model only considering thermal electrons fails. The calculated image terminates at ∼2 mas because it reaches the outer boundary of our simulation domain. For comparison, the best result obtained in previous work is only ~1 mas (*26*), similar to our “thermal-only” model. Moreover, the long-standing puzzle of the observed limb-brightening feature of the jet is clearly reproduced, as shown by Fig. 3 (“Calculation of radiative transfer in the jet” section in Materials and Methods). By contrast, models that only consider thermal electrons or homogeneous distributions of nonthermal electrons produce very weak limb-brightening features (*26*, *28*). In addition, we have examined a test model in which *N*_pl_/*N*_tot_ is fixed to be 0.5, while all other model parameters remain the same with our fiducial model. The jet images predicted by this model have been calculated and are shown in fig. S6. Comparing the (flux contour) results with those presented in Fig. 2, we can see that this model is more similar to the thermal-only model rather than the current-density model because the limb-brightening feature is only present at large distance, and the jet is very short. The total flux densities predicted by our model at 86 and 43 GHz are 1.24 and 2.0 jansky (Jy), in comparison with the observed ranges of 0.95 to 1.59 Jy and 0.99 to 1.33 Jy at these two frequencies, of which 58 and 64% originates from nonthermal electrons. Because the plasma density in our model is normalized to reproduce the flux density at 230 GHz, our model correctly reproduces the flux densities at 230 and 86 GHz but moderately overpredicts that at 43 GHz. This is likely because our model is still too simplified in some aspects.

**Fig. 4.**
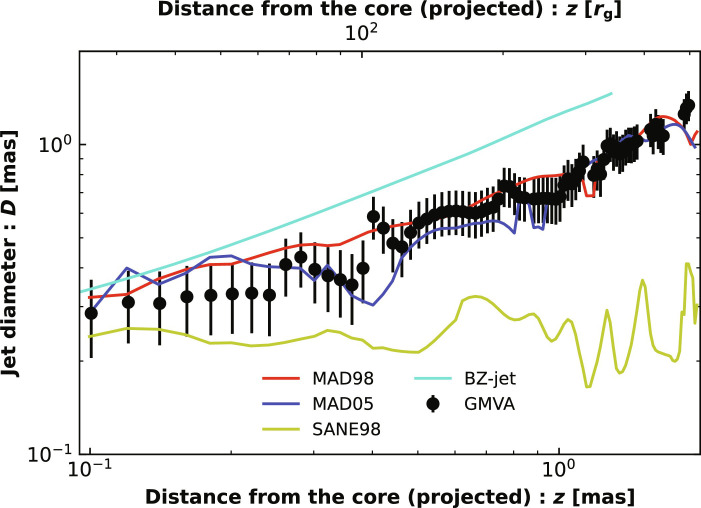
Comparison between the predicted and observed jet width as a function of the projected distance from the core. The observational data are based on stacked GMVA observations at 86 GHz (*64*). The cyan line corresponds to the boundary of the BZ-jet. The fiducial MAD98 model well reproduces the observed jet width throughout the projected jet extent from 0.1 to 2 mas but underpredicts the jet width within 0.1 mas recently measured by Lu *et al.* (*40*). This is likely because the resolution of our simulations is not high enough (“Convergence of numerical resolution” section in Materials and Methods).

To quantify the jet morphology, Fig. 4 illustrates the variation of the jet width as a function of projected distance from the core as predicted by different models (“Calculation of the jet width” section in Materials and Methods) and compares them with observations. The jet width predicted by the MAD98 model agrees reasonably well with observations throughout the entire projected jet extent from 0.1 to 2 mas. This improvement compared to previous works, along with the better match of the length and limb-brightening of the jet, is attributed to our more physical model for the nonthermal electron population. That the jet width is smaller than the boundary between a BZ-jet and disk-jet (cyan line in Fig. 4) indicates that the observed jet corresponds to the former instead of the latter.

Our fiducial model also satisfies the constraints based on the velocity field, jet power, and polarization (text S2). In text S3, we investigate the effects of black hole spin and the accretion mode on the predicted jet image. We find that a MAD and a rapid spin are favored to explain the observations. In some jet models, the emission is dominated by electron-positron pairs within the jet, even close to the horizon scale. The possibility of giving constraint on this mechanism based on our model is discussed in text S4.

Our results bridge the dynamical model of jet formation and observations, demonstrating the viability of the BZ-jet model and magnetic reconnection as the mechanism for electron acceleration in jets. The models will be sharpened in the future by incorporating more detailed particle acceleration by reconnection, obtaining more elaborate electron energy distribution in the jet, and by comparison with observations of higher resolution.

## MATERIALS AND METHODS

### Three-dimensional GRMHD numerical simulation of a hot accretion flow

We have performed numerical simulations in three-dimension by solving the equations of ideal MHD describing the evolution of the accretion flow around a black hole in the Kerr metrics using the GRMHD code Athena++ (*29*, *30*). The code uses a finite-volume Godunov scheme to ensure total energy conservation, with the flux of conserved quantities obtained by solving the Riemann problem at each interface. The commonly used HLLE Riemann solvers are used in our simulations (*31*). The staggered-mesh constrained transport method is applied to satisfy the divergence-free constraint to prevent spurious production of magnetic monopoles. We use the piecewise linear method for spatial reconstruction. It will be interesting to examine the effects of adopting the piecewise parabolic method in the future (*32*).

We perform all our simulations in Kerr-Schild (horizon penetrating) coordinates (*t*, *r*, θ, φ). The radius of the black hole horizon is $r_{H}=\left(1+\sqrt{1-a^{2}}\right)r_{g}$ , with $r_{g}=\frac{GM_{\mathrm{BH}}}{c^{2}}$ the gravitational radius and *a* the spin parameter of the black hole. The determinant of the metric *g* ≡ det (*g*_μν_) = −Σ^2^sin^2^θ, where Σ = *r*^2^ + *a*^2^cos^2^θ (*33*). We use ρ to denote the comoving rest-mass density, and *u*^μ^ as the component of the coordinate-frame four velocity. The equation of state of the gas is *u* = *p*_gas_/(Γ − 1), where the *p*_gas_ is the gas pressure of the comoving mass, *u* is the internal energy of the gas, and Γ is the adiabatic index. We set Γ = 4/3 in our simulation and use unit* r*_g_/*c* to measure the time. The unit we adopt is Heaviside-Lorentz, in which both the light speed and gravity constant are set to be unity, and the sign convention of the metric is (−, +, +, +). The metric is stationary, and the self-gravity of the accretion flow is ignored in our simulations.

There are two accretion modes, SANE and MAD, depending on the magnetization of the accretion flow (*5*, *34*, *35*). We have simulated four models, namely, MAD98, MAD05, MAD00, and SANE98. They denote the MAD accretion flow around a black hole of spin *a* = 0.98, 0.5, and 0.0, and SANE accretion flow around a black hole of spin *a* = 0.98. In all models, the simulation starts with a rotating torus around a black hole. The torus is in a hydrostatic equilibrium state as described by Fishbone and Moncrief (*36*). The inner edge of the torus is at *r* = 40.5*r*_g_, and the radius of pressure maximum is at *r* = 80*r*_g_. We have also added a poloidal magnetic field threading the torus in the way described by Penna *et al.* (*37*). For MAD and SANE, we use two different initial magnetic field configurations. For MAD, we set one poloidal loop threading the whole torus. This leads to rapid accretion and accumulation of a large amount of magnetic flux near the black hole, which eventually impedes the accretion of mass. For a given mass accretion rate, the magnetic flux quickly saturates at a maximum value on the black hole, reaching the MAD state. For SANE, we initially set up a seed field consisting of multiple poloidal loops of magnetic field with changing polarity. This structure facilitates magnetic reconnection and prevents the accumulation of magnetic flux, such that the magnetic field always remains weak, making the accretion flow stay in the SANE mode (*34*, *35*).

To determine the specific magnetic field of the torus, three parameters are required, namely, *r*_start_, *r*_end_ and λ*_B_*. The first two represent the inner and outer edges of the magnetized area, while the last one controls the size of the poloidal loops or equivalently the number of the loops. We use the gas-to-magnetic pressure ratio β ≡ *p*_gas_/*p*_mag_ to normalize the magnetic field. For each loop, it has a minimum value in the equatorial plane. It peaks at the edge of the loop and then drops to the center of the loop. For MAD, we set *r*_start_ = 25 *r*_g_, *r*_end_ = 810 *r*_g_, λ*_B_* = 25 (it just has one loop), and β_min_ = 0.1. For SANE, we set *r*_start_ = 25 *r*_g_, *r*_end_ = 550 *r*_g_, λ*_B_* = 2.5 (it has eight loops), and β_min_ = 0.1 (*a* = 0.98). We have performed test simulations with higher values of β_min_ = 1, 10, 100 and found that the results remain very similar.

We use a static mesh refinement grid, which includes a root grid and additional refined grids in the jet region of interest to the present work. For MAD98, the root grid is 88 × 32 × 16 cells in the radial, polar, and azimuthal directions. We adopt logarithmic spacing, with the ratio $\frac{r_{i+1}}{r_{i}}$ being 1.0827 in the *r* direction. For SANE98, MAD05, and MAD00, the root grid is 110 × 32 × 16 cells in the radial, polar, and azimuthal directions, and the ratio $\frac{r_{i+1}}{r_{i}}$ is 1.0667 in the *r* direction. For MAD98 (SANE98, MAD05, and MAD00), their inner and outer edges are located at 1.1 *r*_g_(1*r*_g_) and 1200 *r*_g_, respectively. The grid in the polar and azimuthal directions are uniform. Because Kerr-Schild coordinates are used, the simulated inner edge is within the black hole horizon.

We use different static mesh refinement for different models. Tables S1 and S2 give the details of the four models. For a refinement region, each additional level means that the grid of this area is refined by a factor of 2 for all directions based on the previous level grid. Note that in ATHENA++, a higher level SMR block has not to be contained within a lower level SMR block. Level 4 is added to improve the resolution of the most important jet region for our modeling. However, to save computation resources, we only add this level in the forward jet region but not in the backward jet region. The last entry in level 3 is added to emphasize this special grid setting. The final grid level for MAD98 is shown in fig. S1. To prevent unphysical (purely numerical) causal contact, our setup ensures that there are at least four cells within the event horizon in all directions for the four models. The final effective resolution upon which our fiducial model is based on 1408 × 512 × 256 for MAD98 and 880 × 256 × 128 for SANE98, MAD05, and MAD00. This effective resolution is achieved within a range of radii, which is most important for our modeling.

Within the BZ-jet region, we have 32 grids at *z* ~ 700 *r*_g_ and 44 grids at *z* ~ 100 *r*_g_ in the θ-direction for MAD98. The number of grids within the BZ-jet region at *z* ~ 100*r*_g_ for SANE98, MAD05, and MAD00 are 15, 24, and 24, respectively.

We use the outflow boundary conditions at the inner and outer boundaries. For the θ and φ direction, we use the polar axis reflective boundary condition and periodic boundary conditions respectively. Because vacuum cannot be handled in numerical simulations, the density and gas pressure floors must be imposed: ρ_min_ = max (10^−2^*r*^−1.5^, 10^−6^), *p*_gas,min_ = max (10^−2^*r*^−2.5^, 10^−8^). We also enforce σ < 100 and γ < 50. Here, σ = 2*p*_mag_/ρ is the magnetization parameter, and γ ≡ α*u^t^*, with the lapse α ≡ (−*g^tt^*)^−0.5^. The former is an additional limitation of the density and gas pressure floors; the latter limits the velocity to prevent the Lorentz factor of the normal frame from becoming too large (*38*).

We run simulations up to *t*_f_ = 40,000 for MAD98, MAD05, MAD00, and SANE98. They correspond to 8.9 orbital periods of the accretion flow at the pressure maximum. The “inflow equilibrium” reached about 80 *r*_g_ for the three MAD models and 30 *r*_g_ for SANE98. Figure S2 shows the distribution in the *x*-*z* plane of several physical quantities for MAD98, while fig. S3 shows the distribution in the *x-y* plane at *z* = 600* r*_g_ of physical quantities for this model. In all models except MAD00, a BZ-jet is successfully produced. The outer boundary of a BZ-jet is defined as the time-averaged surface consisting of magnetic field lines anchored at the event horizon of the rotating black hole with θ = 90°, which also serves as the boundary between the BZ-jet and disk-jet. This is shown by the red lines in each panel of fig. S2. In the present work, we do not perform a detailed analysis of the BZ-jet and disk-jet. Analysis of the BZ-jet can be found in (*3*, *4*), while the analysis of the disk-jet in the case of SANE can be found in (*10*).

We believe that the jets in our simulations have reached a steady state. Detailed virtual particle trajectory analysis indicates that both jet and wind close to the rotation axis are produced at small radii of the accretion flow (*10*). In addition, the velocity of jet material is several orders of magnitude higher than that of the accretion flow; thus, *z*/*v_z_* in the jet is much smaller than the accretion time scale of the accretion flow. So once the innermost region of the accretion flow has reached steady state, as in our case, the jet should also very quickly reach its steady state.

### Convergence of numerical resolution

We first examine the convergence of MHD turbulence driven by magneto-rotational instability (MRI). We follow (*39*) to calculate *Q*_θ_ and *Q*_ϕ_ based on our simulation data

$$Q_{\theta }=\frac{\lambda_{\text{MRI}}}{dx^{\theta }}=\frac{2\pi |v_{\theta ,A}|}{\Omega dx^{\theta }}$$ (1)

$$Q_{\phi }=\frac{\lambda_{\text{MRI}}}{dx^{\phi }}=\frac{2\pi |v_{\phi ,A}|}{\Omega dx^{\phi }}$$ (2)

Here *V*_θ,A_ and *V*_ϕ,A_ are the θ- and ϕ-directed Alfven speeds, and Ω is the angular velocity of fluid. The physical meaning of these two quantities is the number of grid in one fastest growing wavelength of MRI in the θ- and ϕ-directions, respectively. According to Hawley *et al.* (*39*), if *Q*_θ_ > 10 and *Q*_ϕ_ > 20, the resolution of the simulation will be high enough to resolve MRI and give quantitatively converged results for the nonlinear MHD turbulence in the accretion flow. We have calculated these two parameters and found that both are larger than 50 in almost all regions of our simulation domain.

We also need to examine the convergence of current density calculations, especially because in our MHD simulations, dissipation is not explicitly modeled. This issue has been discussed in (*25*). They show that even if the current density does not converge with resolution, the total dissipation rate does.

Last, to examine the convergence of our main results, we have conducted a simulation of MAD98 model with a lower resolution of 352 × 128 × 64. This resolution is lower by a factor of 4 in each direction than our fiducial MAD98 model. We then have examined the convergence in the following two ways. One is that we have compared both the time and ϕ-averaged “basic physical quantities” (i.e., density, temperature, and plasma β) in the two models. The results are shown in fig. S4, indicating reasonable convergence. In addition, using the low-resolution simulation data, we have also produced the jet image at 86 GHz and examined the limb brightening and the jet width. These results are shown in fig. S5. Comparing the results shown in fig. S5 with corresponding results predicted by the high-resolution model, we again find satisfactory resolution convergence. However, note that the limb-brightening feature predicted by the low-resolution model is not as good as that predicted by the high-resolution fiducial model, indicating the role of adopting higher resolution.

In addition to limb brightening, the predicted jet width may also be related with the simulation resolution. We find that our fiducial MAD98 model underpredicts the jet width at *z* < 0.1 mas from the black hole most recently measured by Lu *et al.* (*40*). We think the main reason is that we fail to resolve the limb brightening at this region of the jet, which is likely because our simulation resolution at *r* ≤ 30*r*_g_ is still not high enough (refer to table S1). It will be interesting to test this possibility by increasing the simulation resolution in the future, although the calculation will be very expensive.

### Determination of distributions of thermal and nonthermal electrons

The temperature of thermal electrons in the simulation is determined using the following formula (*14*)

$$\frac{T_{p}}{T_{e}}=R_{\text{low}}\frac{1}{1+\beta^{2}}+R_{\text{high}}\frac{\beta^{2}}{1+\beta^{2}}$$ (3)

Here *T*_p_ and *T*_e_ are the proton and electron temperature, and β is the ratio between the gas and magnetic pressure. We have tested different values of *R*_low_ and *R*_high_ and found that *R*_low_ = 1 and *R*_high_ = 80 provide the best result. These two values are also commonly used in the literature. We note that our value of *R*_low_ is different from that obtained in Event Horizon Telescope Collaboration (EHTC) (*12*), where its value is required to be 10 for the MAD model with a high black hole spin. One possible reason for the discrepancy is that their value is based on an assumption that the observed low polarization of the nuclear region of M87 is due to Faraday rotation internal to the emission region. This assumption is not adopted in our model. In addition, the EHT work focuses on the accretion flow while our work focuses on the jet. The properties of the plasma in the accretion flow and jet, such as the plasma sigma, are notably different thus the electron heating may be different.

The nonthermal electrons accelerated by magnetic reconnection are assumed to be described by the following power-law form

$$\frac{dn_{\text{pl}}}{d\gamma }=N_{\text{pl}}\left(p-1\right)\gamma^{-p},\gamma_{\text{max}}>\gamma >\gamma_{\text{min}}$$ (4)

In the literature, the value of power-law index *p* is often treated as a constant free parameter. In our work, however, we use the most recent results obtained in (*24*). In this work, they present a model for determining the value of *p*, with the processes of particle injection, acceleration, and escape included. They use the results of a series of first-principle fully kinetic simulations to calibrate a couple of model coefficients. This model not only can successfully reproduce the simulation results but also can predict the power-law index *p*. The value of *p* is described by

$$p=\frac{1}{\sigma_{x}+0.2[1+\text{tanh}\left(b_{g})\right]}+0.04\text{tanh}\left(b_{g}\right)\sigma_{x}+1.7b_{g}+2.1$$ (5)

Here *b*_g_ is used to describe the guide field* B*_g_ =* b*_g_ *B*_0_, *B*_0_ is the reconnection magnetic field, $\sigma_{x}=B_{0}^{2}/w$ , with $w=\left(\rho +P_{\text{gas}}+u_{\text{gas}}+B_{0}^{2}\right)$ is the enthalpy density. In our work, we consider the magnetic field from our GRMHD simulation as the total magnetic field, so $B=\sqrt{B_{0}^{2}+B_{g}^{2}}$ . We set* b*_g_ = 0.5. We have performed several tests and found that our results are not sensitive to its value. The distribution of the value of *p* is shown by fig. S7.

PIC simulations of particle acceleration by reconnection find that the exact value of γ_min_ depends on many parameters such as β and the guide field and is still poorly determined (*41*). Different ways are adopted in the literature to set the value of γ_min_. Özel *et al.* (*42*) and Yuan *et al.* (*43*) require that the number density of nonthermal electrons must be equal to that of thermal electrons at γ_min_. Dexter (*44*) treats it as a free parameter, while Chatterjee (*45*) ties γ_min_ to the peak of the thermal distribution, namely, γ_min_ = *u*_th_/*m*_e_*c*^2^ + 1 ≈ 3k*T*_e_/*m*_e_*c*^2^ + 1 for relativistic electron temperature. Here, *u*_th_ is the energy density of thermal electrons. In our work, we set a higher value, γ_min_ = 10k*T*_e_/*m*_e_*c*^2^ + 1, at which particle energy is well suprathermal. The value of γ_max_ is not important for the calculation of radiation if the value of *p* is not too small as in our case. We have tested two values of γ_max_, 10^5^ and 10^6^, and the differences of results are found to be negligible.

For our problem, the most important quantity to determine is the amount of nonthermal electrons and their spatial distribution. Many different ways have been adopted to specify this parameter in the literature. In some works, its value is determined by assuming that the energy density of nonthermal electrons is a constant fraction of the energy density of thermal electrons (*42*, *43*, *46*, *47*) or magnetic field (*44*, *48*). The dependence of acceleration efficiency in magnetic reconnection on the plasma β and magnetization parameter σ is investigated by PIC simulations in (*22*, *24*), and the result is adopted in (*45*) and (*49*) in their studies of flares from accretion flows.

In our work, we assume that the electrons are accelerated by magnetic reconnection. Following (*25*), we assume that the acceleration rate is proportional to the square of the local three-current density measured in the frame of the plasma, **J**^2^. We found that the radiative time scale of accelerated electrons is different at different distances in the jet and can be shorter than the local dynamical time scale; therefore, radiative cooling should be included when we try to obtain a steady energy distribution of nonthermal electrons. Given the above analysis, the steady number density of nonthermal electrons *N*_pl_ is determined by solving the following equation

$$\eta \frac{v_{A}}{r_{z}}\left(N_{\text{tot}}-N_{\text{pl}}\right)\frac{J^{2}}{J_{0}^{2}}=\frac{N_{\text{pl}}}{\tau_{\text{cool}}}$$ (6)

Here, *N*_tot_ is the total number density of electrons, including thermal and power-law electrons, which is taken directly from our simulations; η is a dimensionless parameter that controls the efficiency with which currents accelerate electrons into the power-law distributions, with its value being given in table S3 for various models; and *v*_A_ is the Alfven speed, which determines the magnitude of the reconnection inflow speed (*20*, *21*). The difference between the reconnection inflow speed and *v*_A_ is absorbed in η. *r*_z_ denotes the typical length scale of the reconnection region. Within the jet region, the reconnection is likely driven by the strong perturbation of the magnetic field lines caused by the magnetic eruption in the accretion flow (see the “Physical origin of magnetic reconnection in the jet” section in Materials and Methods for the discussion), so *r*_z_ should be the scale of jet width. Motivated by the observed jet width as a function of jet distance (refer to Fig. 4), we set *r*_z_ = *z*^1/3^. Outside of the jet region, we simply set *r*_z_ = *r*. The boundary between these two scalings of *r*_z_ should be beyond the BZ-jet boundary because the perturbation to the field lines caused by the magnetic eruption originates from the innermost region of the accretion flow, and it should propagate mainly in the polar region along the field lines. In our calculation, we therefore choose a line described by *R* = 2 + 2 × *z*^0.7^ as the boundary of the two different scalings. This boundary well mimics the shape of a magnetic field line beyond the BZ-jet boundary. The discontinuity at the boundary produces the sharp feature of *N*_pl_/*N*_tot_ presented in Fig. 1B. We have performed tests by adopting different boundaries for different scalings and by adopting the same scaling in the whole region. We have confirmed that the discontinuity here will not affect our main results. Especially, the limb brightening of the jet remains unchanged. τ_cool_ is the local radiative cooling time scale of nonthermal electrons. In general, it depends on electron Lorentz factor γ. In the present work, given that the value of power-law index determined by Eq. 5 is usually large, ∼ 4 − 6 (refer to fig. S7), we adopt a simplification by only considering electrons with γ_min _when we estimate τ_cool_. This simplification is similar to that adopted in “model D” in (*25*). **J** is the local three-current density, which is calculated following (*50*)

$$J^{i}=\partial_{j}F^{\mathrm{ij}}+\Gamma_{j\lambda }^{i}F^{\mathrm{ij}}$$ (7)

where *F*^μν^ is the electromagnetic tensor, $\Gamma_{\alpha \beta }^{\lambda }$ is the Christoffel symbols, and *i* equals to *t*, *r*, θ, and ϕ. The characteristic three-current density $J_{0}^{2}\equiv c^{2}P/r^{2}$ , with *P* and *r* being the gas pressure and spherical radius, respectively (*25*).

### The physical origin of magnetic reconnection in the jet

Some works have suggested that the jet is subject to current-driven kink instability, which can disrupt magnetic field lines and trigger the magnetic reconnection (*51*–*53*). According to the Kruskal-Shafranov (KS) criterion, cylindrical configurations in which the toroidal component of the magnetic field dominates are unstable to the *m* = 1 kink mode. The classical KS criterion is extended when the relativistic field line rotation is taken into account (*54*). Jets are unstable only if both the KS criterion and the condition ∣*B*_ϕ_/*B*_p_∣ > *R*Ω/*c* are satisfied. Here, *R* is the cylindrical radius of jet, Ω is the angular velocity of the field lines, and *c* is the speed of light. We have evaluated the values of *B*_ϕ_ and *B*_p_ in the comoving fluid frame and found that these two values are overall comparable to each other, although strong spatial fluctuation exists; thus, the jet is not subject to kink instability. This is especially true in the small distance in the jet where the poloidal component of the magnetic field is dominant. However, in that place, nonaxisymmetric features of the current density distribution are found to be as strong as in other distances, as we will show later.

We then consider another possibility; that is, the reconnection in the jet is caused by magnetic eruption in MAD (*4*, *15*–*19*). MHD numerical simulations of MAD have found that the poloidal field will be carried inward by the accretion flow and accumulate on the black hole horizon. When the field pressure overcomes the dynamic pressure of the accreting gas, the magnetic field quasi-periodically erupts. The eruption occurs in the form of low-density, magnetically dominated medium fountained outward from the black hole. This will produce a strong perturbation to the field lines, and the perturbation should propagate vertically outward and trigger magnetic reconnection in the jet. In addition to this mechanism, the high-resolution simulation by Ripperda *et al.* (*18*) indicates that the eruption is accompanied by reconnection in the accretion flow near the horizon, which transforms the horizontal field to a vertical field that is then ejected outward vertically and also triggers reconnection in the jet region.

To examine this possibility, we have performed several tests. Because the eruption is found to be nonaxisymmetric, the distribution of current density in the *x* − *y* plane of the jet caused by this mechanism should be nonaxisymmetric. Using the MAD98 simulation data, we have calculated the distribution of current density and did find a nonaxisymmetric current density distribution, as shown in fig. S8. Next, because eruption events occur in MAD but not in SANE, we should expect that the nonaxisymmetric feature should be much stronger in MAD than in SANE. To examine this point, we have performed a power spectrum analysis of the magnitude of the current density **J** by performing the volume-averaged Fourier transform following the approach presented in (*55*)

$$f\left(m,k\right)=\frac{1}{V_{\text{cl}}}{∭}_{V_{\text{cl}}}|J|e^{i\left(m\phi +\mathrm{kz}\right)}\text{rdrd}\phi \mathrm{dz}$$ (8)

where ∣**J**∣ is the magnitude of **J** and *V*_cl_ is the cylindrical volume that encloses **J**

$$V_{\text{cl}}=\int_{r_{a}}^{r_{b}}\int_{0}^{2\pi }\int_{z_{a}}^{z_{b}}\text{rdrd}\phi \mathrm{dz}$$ (9)

The quantity *f*(*m*, *k*) is a function of the azimuthal mode number *m* and the axial wave number *k* = 2π/λ (λ is the characteristic wavelength). The power spectrum is calculated as

$$|f\left(m,k\right){|}^{2}={\left\{\text{Re}\left[f\left(m,k\right)\right]\right\}}^{2}+{\left\{\text{Im}\left[f\left(m,k\right)\right]\right\}}^{2}$$ (10)

$$\text{Re}\left[f\left(m,k\right)\right]=\frac{1}{V_{\text{cl}}}{∭}_{V_{\text{cl}}}|J|\text{cos}\left(m\phi +\mathrm{kz}\right)\text{rdrd}\phi \mathrm{dz}$$ (11)

$$\text{Im}\left[f\left(m,k\right)\right]=\frac{1}{V_{\text{cl}}}{∭}_{V_{\text{cl}}}|J|\text{sin}\left(m\phi +\mathrm{kz}\right)\text{rdrd}\phi \mathrm{dz}$$ (12)

The typical wavelength can be estimated by the product of the jet speed and eruption period, which are roughly speed of light and 1000 *r*_g_/*c*, respectively (refer to fig. S9 for the eruption period). This means that the wave number κ = 2π/λ ≪ 1; thus, we simply set *k* = 0 in our calculations. The time evolution of the power spectrum for *m* = 1 and *m* = 0 for MAD98 and SANE98 models at *z* ∼ 120*r*_g_ and *z* ∼ 600*r*_g_ in the jet are shown in the two panels of fig. S9. We can see from the figure that, in both panels, the *m* = 1 mode power (normalized by the *m* = 0 mode power) in the case of MAD98 is much larger than SANE98, consistent with our expectation. We note that although at small *z* (∼120*r*_g_) where the poloidal magnetic field component is much larger than the toroidal component, thus the kink instability is expected to be absent, the result still remains the same, indicating again that kink instability is not the physical mechanism of driving reconnection. In addition, the typical time scale of the variability of mode power is roughly consistent with the variability time scale of the magnetic flux threading the horizon (*56*), again consistent with the magnetic eruption scenario. In the case of SANE98, we do not expect the existence of magnetic eruption, but we still find some power from fig. S9, although it is weak. We speculate that the power in this case arises from other mechanisms, such as some kinetic instabilities driven by the interaction between the jet and its ambient medium (*57*) or velocity shear close to the jet boundary (*58*).

### Calculation of radiative transfer in the jet

Because the plasma thermodynamics predicted by any energy conserving simulations is unreliable in highly magnetized region, in our calculation of radiation from jet, the emission originating from the highly magnetized region (σ > σ_cut_) is excluded, as usual. The value of σ_cut_ is highly uncertain, and different values are adopted in the literature (*26*, *28*, *59*). In many cases, a low value of σ_cut_ = 1 is adopted in the study of radiation from SANE. However, because the jet region is highly magnetized, higher values are often adopted for jets. For example, a fiducial value of σ_cut_ = 25 is adopted in (*28*), while 3 ≤ σ_cut_ ≤ 5 is favored in (*26*). Here, we have tested four different values σ_cut_ = 1, 3, 5, 6, and 10. We find that the best results are achieved when σ_cut_ = 5 for our fiducial MAD98 model, and thus, we choose this value. In text S1, we have studied the effects of choosing different boundaries of the radiation region in the jet and found that our results are not very sensitive to the choice of the boundary. For the choice of a notably higher value of σ_cut_, the predicted total flux density at 86 GHz would be too high compared to observations (we use the observed 230 GHz flux density to normalize our accretion rate). In addition, the predicted jet width would be too narrow because the radiation would concentrate more toward the jet axis. The results would go to the other extreme if a notably lower σ_cut_ were adopted.

Because our GRMHD simulation is density scale-free, we need to normalize the density and magnetic field of the simulations. We do this by choosing a density so that the accretion can reproduce the observed radio flux density of 0.5 Jy at 230 GHz obtained by EHT for M87 in the field of view of 100 μas (*60*). The mass of the black hole of M87 is adopted to be 6.5 × 10^9^*M*_⊙_ (mass of the Sun) (*61*). The obtained mass accretion rates for difference models are listed in table S3. We use IPOLE, which is a public ray-tracing code for covariant, polarized radiative transport, to calculate the image of the jet corresponding to various models (*27*). Note that in our calculations of images, the contributions of all plasma in the simulation domain, including the accretion flow and wind launched from the accretion flow, have been included.

For MAD98, we have randomly chosen 50 different snapshots from the simulation data after the simulation has achieved steady state and calculated their corresponding images. The viewing angle of M87 is 163°, and the distance to the black hole is 16.8 Mpc. The field of view of our calculated image is [−2000, 2000] μas both in *x* and *y* directions, and the resolution of the image is 500 × 500. After completing the radiation transfer calculation, we rotate the output image by 108° counterclockwise to make the orientation of the jet consistent with the observed position angle of about 288° (*62*). We have added in the images some flux density contours in the following way. Taking the 86 GHz image as an example, denoting the peak flux density of the predicted image as *S*_predict_ Jy per beam, the value of each contour in the image is $[-0.47,\;0.47,\;0.47\times \sqrt{2},\;0.47\times 2,\;\ldots ]\times 10^{-3}S_{\text{predict}}/S_{\text{obs}}$  Jy per beam, i.e., increasing by factor of $\sqrt{2}$ for two adjacent contours until the peak flux density is reached, as in the observed image. Here, *S*_obs_ (=0.560 Jy per beam) and 0.47 × 10^−3^ Jy per beam are the peak and the lowest flux density of the observed image, respectively. In this way, we ensure to have the same number of contours in both the theoretical and observed images, so that the dynamical ranges of flux density in the theoretical and observed images are the same.

### Calculation of the jet width

We calculate the width of the jet following the same approach of defining the jet width in the analysis of observations (*63*). That is, based on the calculated jet image, we slice the jet in the direction perpendicular to the jet axis and fit the flux-density profile with one or three Gaussians. The jet width is then defined as the deconvolved full width at half maximum of the Gaussian profile or the distance between the two outermost Gaussians.

## Supplementary Materials

### This PDF file includes:

Supplementary Text Figs. S1 to S16 Tables S1 to S3 References
