# Supplementary material for: Modeling the inner part of the jet in M87: Confronting jet morphology with theory
Source: Sci Adv. 2024 Mar 22;10(12):eadn3544. doi: 10.1126/sciadv.adn3544 (PMC12697568; doi:10.1126/sciadv.adn3544)
Supplement: Supplementary file 1 — Supplementary Text Figs. S1 to S16 Tables S1 to S3 References [file sciadv.adn3544_sm.pdf]

Science Advances
AAAS

Supplementary Materials for

Modeling the inner part of the jet in M87: Confronting jet morphology with theory

Hai Yang *et al.*

Corresponding author: Feng Yuan, fyuan@fudan.edu.cn

Sci. Adv. **10**, eadn3544 (2024)
DOI: 10.1126/sciadv.adn3544

This PDF file includes:

Supplementary Text
Figs. S1 to S16
Tables S1 to S3
References

Supplementary Text

S1 The effect of choosing different boundaries of the emission region in the jet

In our fiducial model, we set $\sigma_{cut} = 5$ to determine our boundary of the radiation region in the jet. In this section we examine the effects of choosing different boundaries.

We have first tried different values of σ_{cut}. Fig. S10 shows the predicted images at 86 GHz with $\sigma_{cut} = 3$ & 6, together with the image predicted by the fiducial $\sigma_{cut} = 5$ model. We can see from the figure that three images are very similar to each other. We have also compared the predicted jet width and found they are also similar. The similarity implies that our results are not very sensitive to the choice of σ_{cut}. Physically, the limb-brightening is because of the combination effects of the magnetic field distribution, the spatial distribution of nonthermal electrons, and the Lorentz factor of the jet. From the middle panel of Fig. 1, we can see that the region of large N_{pl}, where we may expect most of the radiation originates, deviates from the $\sigma_{cut} = 5$ line. This perhaps explains why the results are not very sensitive to the value of σ_{cut}. But we note that the results will be notably different if the value of σ_{cut} deviates from $\sigma_{cut} = 5$ too much.

Usually, the motivation of choosing a value of σ_{cut} to determine the radiation region is that we believe the thermodynamics outside of this region obtained in the simulation is no longer reliable. In this case, we should ensure that no matter escapes from this "unreliable'' region and enters the radiation region. To examine this issue, we have compared the streamline and the $\sigma_{cut} = 5$ line, as shown by Fig. S11. We can see from the figure that the $\sigma_{cut} = 5$ line is very close to the streamline, the two lines almost coincide with each other in most of the region.

Alternatively, instead of using σ_{cut} to determine the boundary of the radiation region, we have used the streamline shown in Fig. S11 as the boundary of the radiation region in the jet. Fig. S12 shows the predicted image at 86 GHz in this case. By comparing this result with Fig. 2, we can see that they are very similar.

S2 Additional observational constraints on the jet models: velocity field, power, and polarization of the M87 jet

Multiwavelength VLBI and optical observations provide both subluminal and superluminal features of the proper motion of the jet in M87. The observed velocity field, shown by the dots in Fig. S13, provides a constraint on the jet model (*66*). We examine our simulated velocity and compare with observations. The Lorentz factor in our simulation is calculated by $\Gamma = \sqrt{-g_{tt}}u^t$, which is measured in Boyer-Lindquist coordinates. From the relation between the Lorentz factor Γ and velocity β_v, $\Gamma \equiv (1-{\beta_v}^2)^{-1/2}$, we can calculate the predicted velocity β_v of the jet plasma. The velocity obtained from observation is the apparent speed of the moving components in the jet, β_{app}. The intrinsic speed β_v is calculated using the equation $\beta_v = \beta_{app}/(\beta_{app}cos\theta_v + sin\theta_v)$. The comparison of the value $\Gamma\beta_v$ between simulation and the observation is shown in Fig. S11. We can see that the predicted velocity is consistent with observations.

The second constraint is the jet power. Due to the physical uncertainties in the models used to estimate the jet power and the wide range in length and timescales probed by the observations, a wide range of powers is obtained, from $10^{42} erg\ s^{-1}$ to $10^{45} erg\ s^{-1}$ (62). We calculate the predicted jet power following Ref. (*4*), $P_{BZ} = \frac{\kappa_0}{4\pi c}\Omega_H^2\Phi_{BH}^2 f(\Omega_H)$, where κ_0 is numerical constant that depends on the geometry of the magnetic field and we adopt κ_0=0.044; $\Omega_H = ac/(2r_H)$; Φ_{BH} is the absolute magnetic flux threading one hemisphere of the black hole event horizon; $f(\Omega_H)$ is a modifying factor for high spin a, which is $f(\Omega_H) \approx 1 + 1.38(\Omega_H r_g/c)^2 - 9.2(\Omega_H r_g/c)^4$. The power of BZ-jet calculated in MAD98, MAD05, and SANE98 are $6.3\times 10^{43} erg\ s^{-1}$, $3.1 \times 10^{43} erg\ s^{-1}$, $9.6\times 10^{42} erg\ s^{-1}$, respectively. All are within the observed range.

The third constraint is polarization. High angular resolution polarimetric observations of the nucleus of M87 are performed using the Very Long Baseline Array at 24 GHz and 43 GHz, and polarization structures within ~0.7 (at 43 GHz) -1 (at 24 GHz) mas around the core region are obtained (*73*). The average linear polarization degree is about 2-3%. We have calculated the polarization degree predicted by our fiducial MAD98 model using the IPOLE code. In the calculation, we first get the Stokes I, Q, U and V at 43GHz, then convolve each component with the beam of $0.38 \times 0.17\, mas$ at $-10°$. The calculation result for the central nuclear region of M87 is shown in Fig. S14.

Comparing this result with that shown in Ref. (*73*), we can see that the predicted polarization degree is notably higher than the observed value. The reason is because our calculation only takes into account the depolarization due to the plasma within our simulation domain, i.e., $r \lesssim 1000 r_g$ from the black hole. The RM due to the plasma in this region is small because of the small inclination angle and the low gas density close to the jet axis. From the detailed theoretical modeling presented in Ref. (*13*), the RM contributed by the plasma outside the simulation domain, mainly the wind launched from the hot accretion flow in M87, can be as large as ≥ $10^5\, rad/m^2$; thus strong depolarization is expected there. This prediction is consistent with the result obtained by the VLBA observations (*74*), which show that the RM is of external origin and larger than $10^4\, rad/m^2$ at a de-projected distance of as large as $10^4 r_g$. The detailed calculation of the depolarization by this part of plasma is beyond the scope of the present paper. The high polarization degree we obtain therefore implies that our theoretical prediction is not in conflict with observations.

S3 Images of jet predicted by other accretion modes and black hole spin

To show the effects of accretion mode and black hole spin, we have calculated the images corresponding to other three models, i.e., MAD05, MAD00, and SANE98. We have searched 30 (for MAD05), 20 (for SANE98), and 20 (for MAD00) snapshots from their simulation data after the models have reached their steady states and calculated their images. The representative results are shown in Fig. S15. For MAD05 and SANE98, the predicted jet extends along the jet axis up to 2 mas, similar to MAD98. However, both models fail to reproduce the limb-brightening feature. Moreover, the predicted jet width by the SANE98 model is too small compared to observations, as shown by Fig. 4.

To understand the reasons, we show in Fig. S16 the two-dimensional spatial distributions of N_{pl}/N_{tot}. For all three models, we find that, at distances not too far away from the black hole, say $z < 400r_g$, the large value of N_{pl}/N_{tot} (thus high number density of accelerated nonthermal electrons) are distributed in a rather broad layer, not as narrow as in the case of MAD98. This may be the reason for the absence of limb-brightening in their predicted images. For SANE98, different from MAD98 and MAD05, the value of N_{pl}/N_{tot} is also very high at the region very close to the jet axis. This explains why the jet width predicted by SANE98 is too small, as shown in Fig. 4. For MAD00, we find from the figure that the value of N_{pl}/N_{tot} close to the black hole is notably smaller than the other three models. This implies there are fewer nonthermal electrons in the jet and may explain why almost no elongated jet-like structure is seen in the middle panel of Fig. S15. The results of the spatial distribution of N_{pl}/N_{tot} in all four models, including their differences among them, should be caused by their specific configurations of the magnetic field lines, which in turn must be due to the differences of accretion mode and black hole spin.

S4 Is it possible to put constraints on the pair cascade model?

In addition to the electron-proton model presented in the present paper, there are alternative electron-positron models in the literature for the emission of the jet in M87 (*75-79*). In these models, pairs are produced by photon-photon collision processes and are accelerated by unscreened electric fields in the magnetosphere of the rotating black hole. The accelerated particles Compton up-scatter background photons, which collide with other background photons and produce a pair cascade. The radiation of these pairs could be responsible for the observed radiation of the jet. Such a process cannot be evaluated using ideal GRMHD simulation models as presented in this work. But whether our model can present some constraints on this mechanism?

There exists a critical charge number density in the magnetosphere below which a strong electric field forms and electrons can be accelerated and consequently a cascade of pair production occurs, i.e., the Goldreich-Julian density, n_{GJ}. Detailed calculations have shown that the number density of pairs produced by the pair cascade mechanism in the spark gap is ~ $10^3 n_{GJ}$ (*76*). In the following, we estimate the value of n_{GJ} and compare it with the number density obtained in our model.

Instead of directly comparing the number density, we compare the number flux. The underlying assumption is that, the pairs produced in the magnetosphere should be advected into the jet and the number flux should be conserved. The number flux of electrons corresponding to the Goldreich-Julian density is roughly estimated to be:

$$F_{GJ} \sim n_{GJ} {r_g}^2 v_r, \qquad (13)$$

where $n_{GJ} = \frac{\Omega B}{4\pi e c}$, $\Omega=\frac{a c^3}{4 G M_{BH}}$, r_g is assumed to be the radius of the gap, v_r is the typical radial velocity of the pairs in the gap, and e is the electron charge. On the other hand, in our model the number flux of electrons at a large radii r within the BZ-jet is estimated to be:

$$F_e \approx 2\pi \int_{\theta_{BZ}} v_r n_e \Gamma r^2 sin(\theta) d\theta, \qquad (14)$$

where $n_e = n_i = \rho/m_p$, Γ is the bulk motion Lorentz factor of the jet, $v_r \sim c$ is the typical radial velocity at r in the jet.

We have calculated the above two integrals using the values obtained from ϕ-averaged values of our fiducial MAD98 model. We find that n_{GJ}=$4.1 \times 10^{-7} cm^{-3}$, $F_{GJ} \approx 10^{33} s^{-1}$. At $r{\sim}500\, r_g$, $F_e \approx 10^{45} s^{-1}$. So we have $\frac{F_e}{F_{GJ}} {\sim} 10^{12}$. This value roughly agrees with Ref. (*79*). Such a large ratio suggests that pair cascades in the black hole magnetosphere are likely not able to provide enough electrons to produce the observed radio flux of the jet in M87.

In addition to the number flux, another constraint comes from timescale. The radiative timescale of pairs produced in the black hole magnetosphere should be long enough so that they can propagate into a large distance of the jet and produce the radio emission there. The dynamical time is roughly $t_{dyn} = 1.6 \times 10^7 (\frac{z}{500\, r_g})\, s$, while the radiative timescale at the magnetosphere is roughly $t_{cool} = \frac{9\, m_e{}^3 c^5}{4e^4 B^2 \gamma_{min}} = 1.4 \times 10^3 (\frac{B}{74G})^{-2} (\frac{\gamma_{min}}{100})^{-1}\, s$. The radiative timescale is much shorter than the dynamical timescale, which is another evidence against the pair produced in the black hole magnetosphere to be responsible for the observed radiation in the jet.

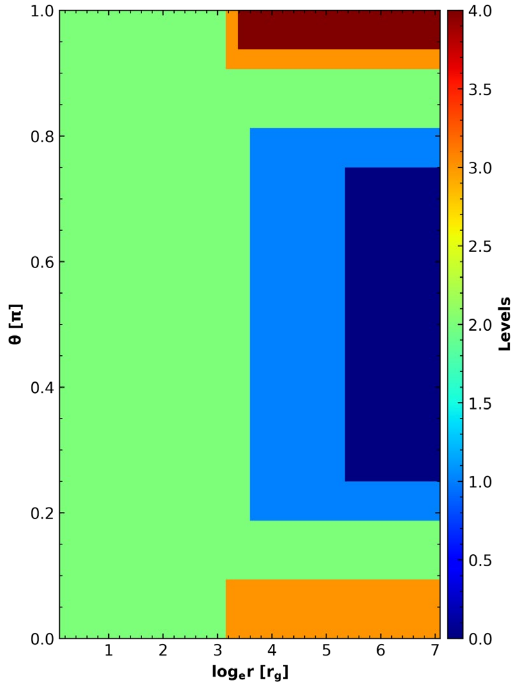

Fig. S1. The final grid level for MAD98 in the $log(r) - \theta/\pi$ plane.

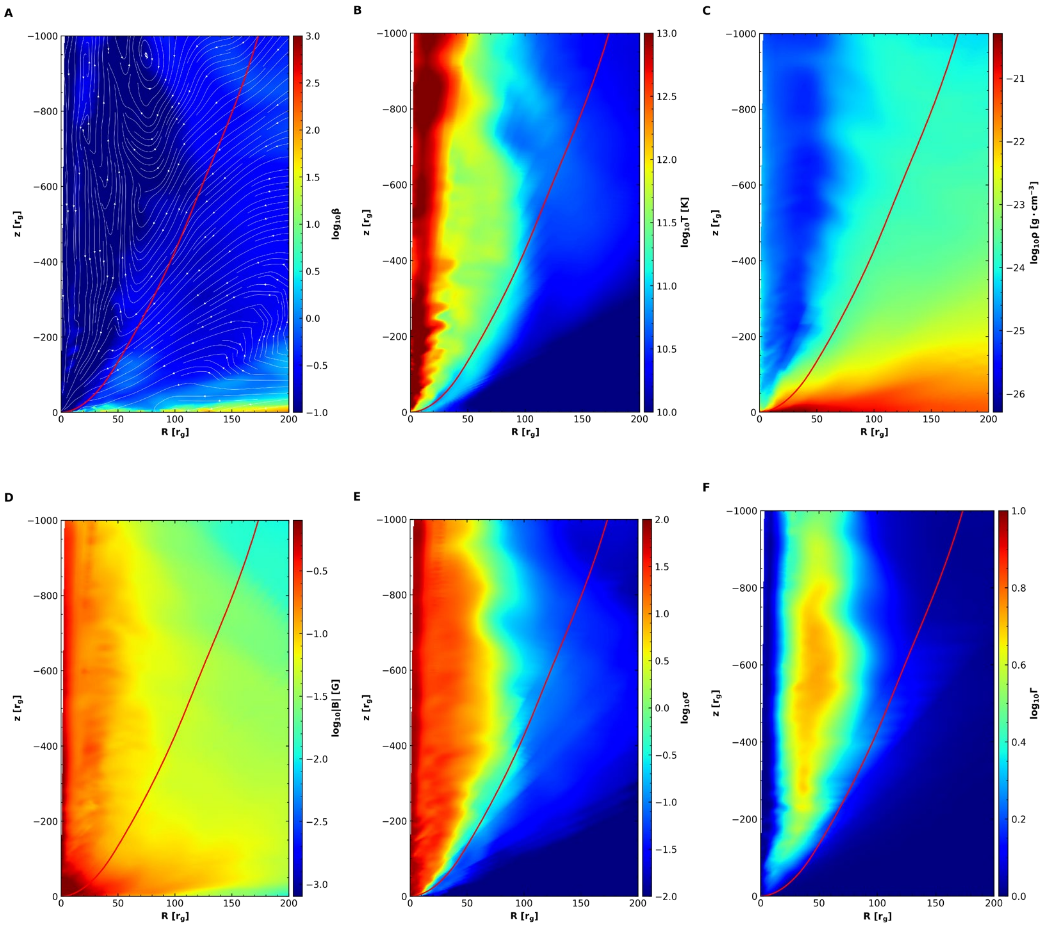

Fig. S2. The ϕ-averaged two-dimensional distribution of different physical quantities in R-z plane. They are the (**A**) plasma β, (**B**) temperature, (**C**) number density, (**D**) magnetic field strength, (**E**) magnetization parameter, and (**F**) Lorentz factor of MAD98 at a simulation time of t=27,400, the same time as Fig. 1. The white curves in the top-left panel denote magnetic field lines. The red lines in all panels denote the ϕ-averaged boundary of the BZ-jet.

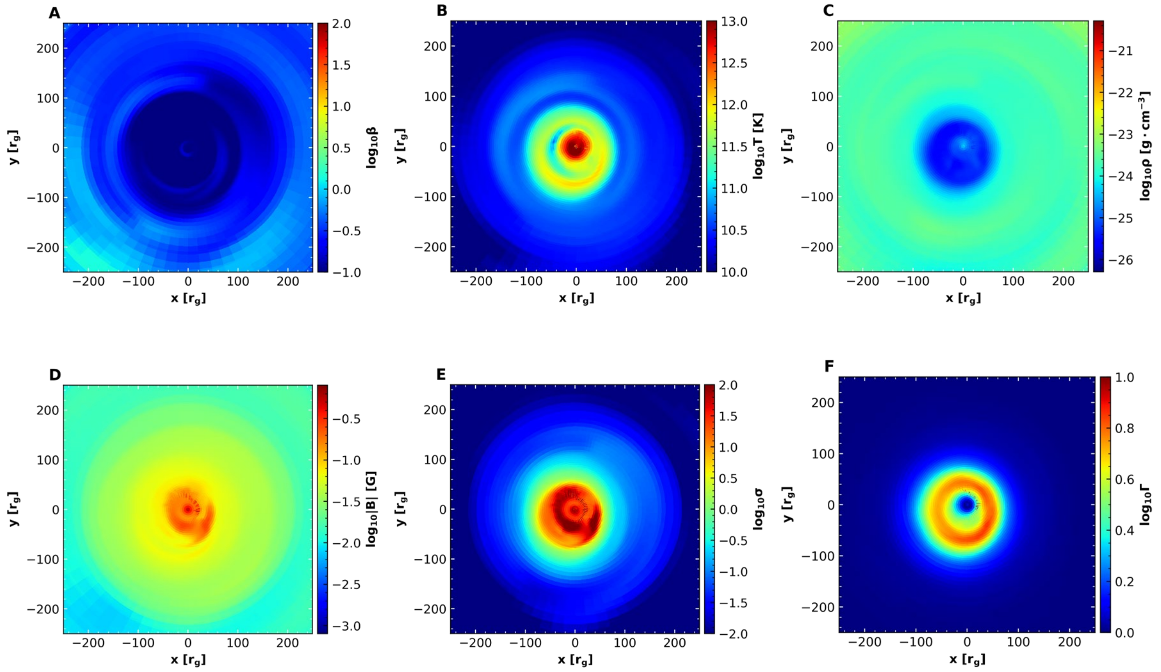

Fig. S3. The two-dimensional distribution in the $x-y$ plane at $z=600r_g$ of different physical quantities. (**A**) plasma β, (**B**) temperature, (**C**) density, (**D**) magnetic field strength, (**E**) magnetization parameter, and (**F**) Lorentz factor of MAD98 at a simulation time of t=27,400, the same time as Fig. 1.

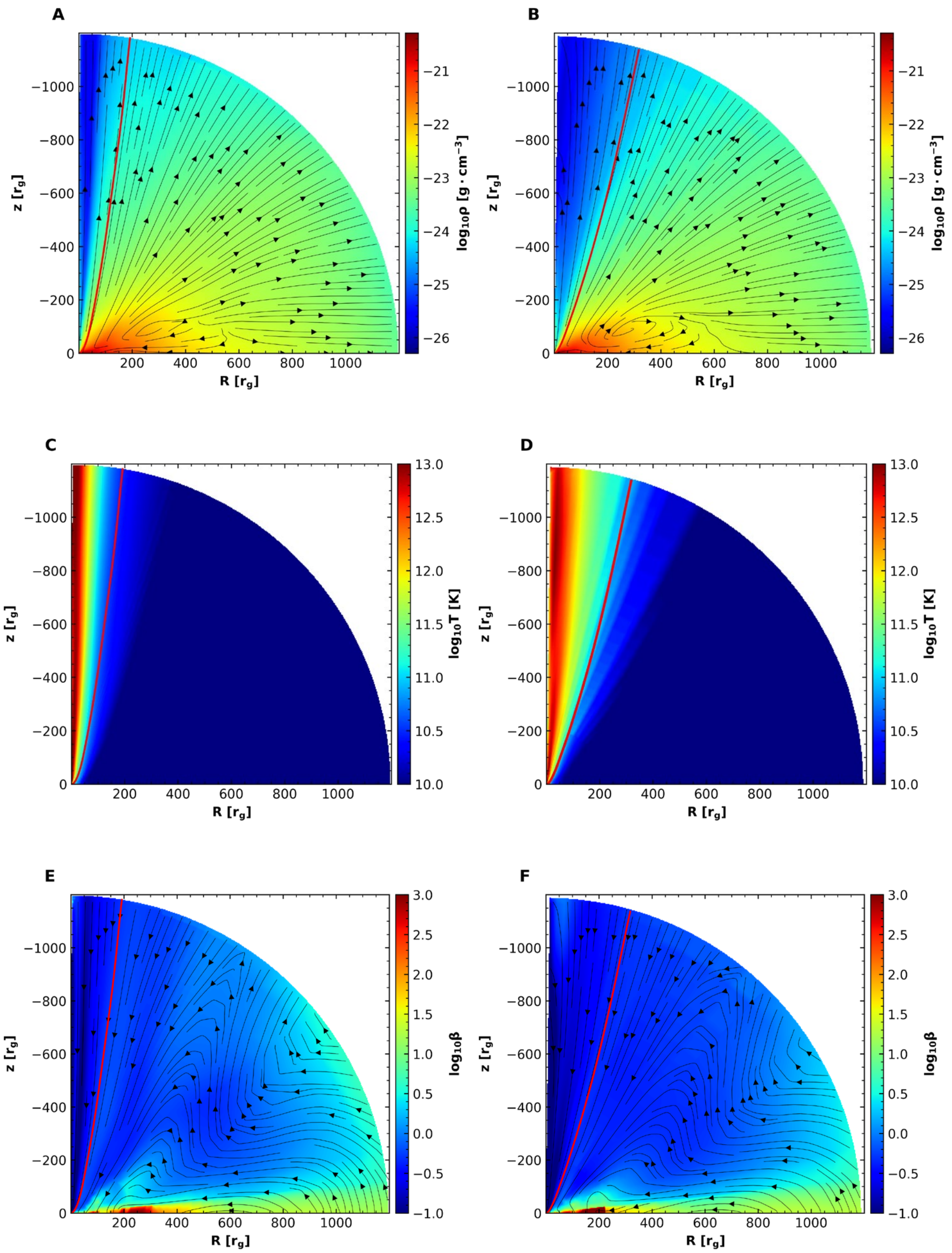

Fig. S4. Comparison of time and ϕ-averaged physical quantities at high and low resolutions. (**A** and **B**) density, (**C** and **D**) temperature, and (**E** and **F**) plasma β for high (left) and low (right) resolution simulations of MAD98. The red line in all panels denotes the ϕ-averaged boundary of the BZ-jet. The black curves with arrows in the top panel denote velocity field lines, the black curves with arrows in the bottom panel denote magnetic field lines.

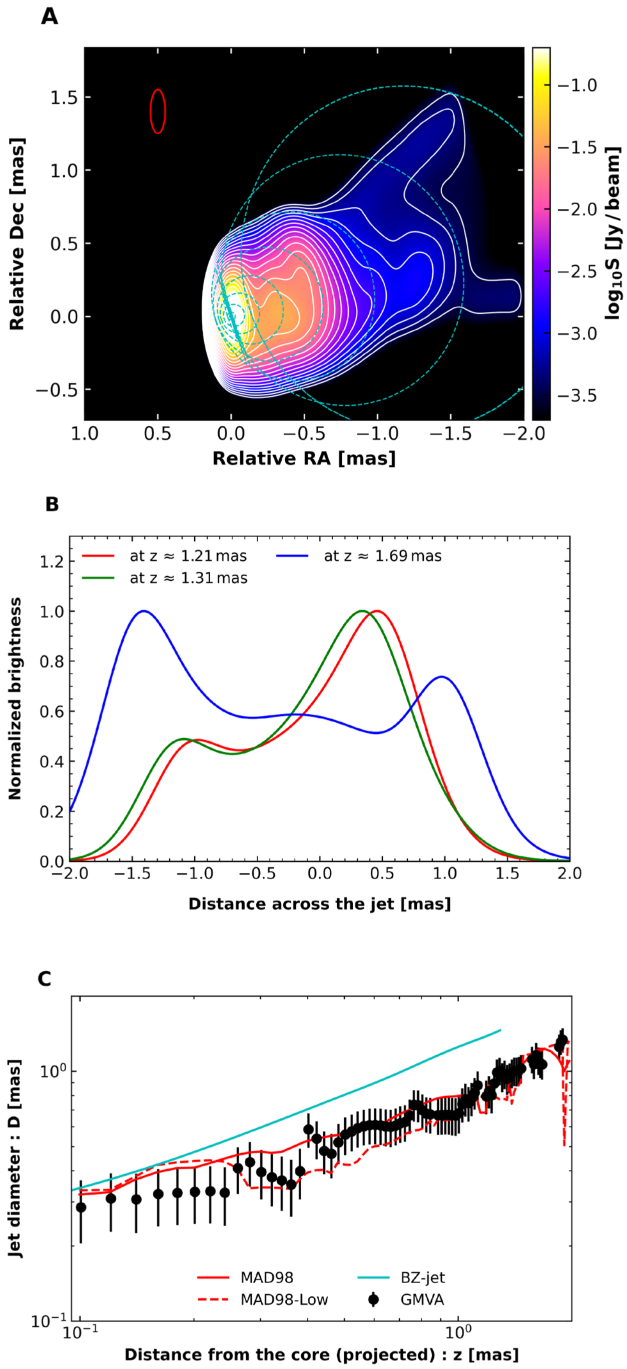

Fig. S5. The predictions of the low-resolution MAD98 model. (**A**) The images at 86 GHz. (**B**) limb-brightening feature. (**C**) the jet width. The dotted circles in the top panel denote the boundary of the BZ-jet.

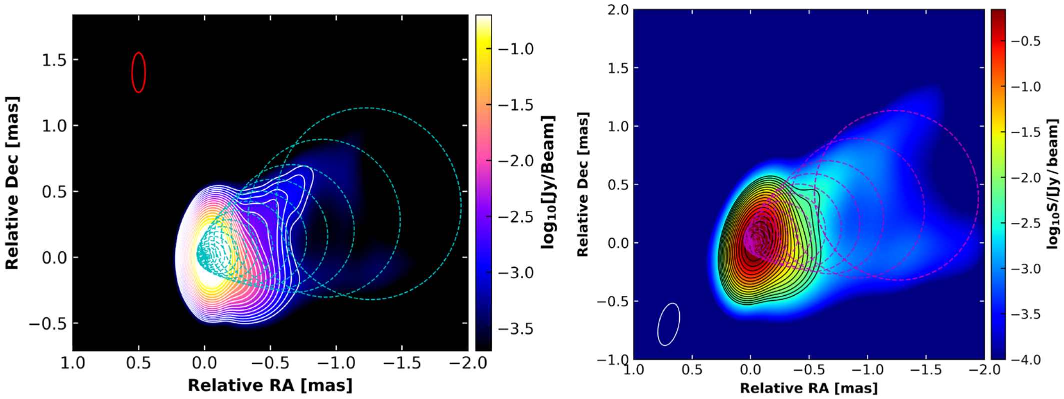

Fig. S6. Images predicted by a N_{pl}/N_{tot} = 0.5 test model. (**A**) 86 GHz and (**B**) 43 GHz. All other model parameters are the same with our fiducial "current density" model. Comparison with Fig. 2 indicates that this model is more similar to the "thermal-only'' model rather than the "current density'' model.

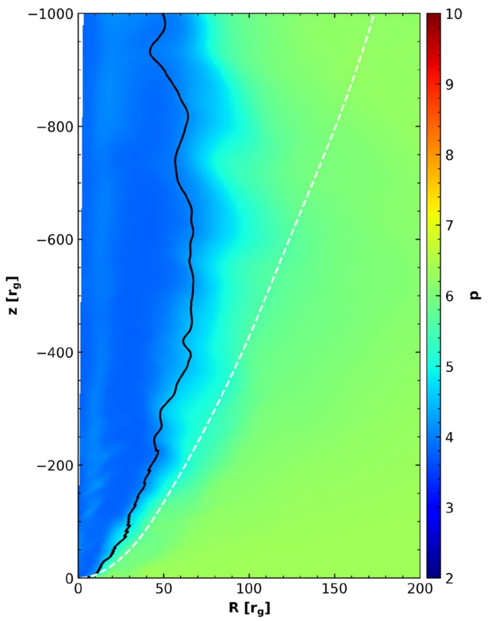

Fig. S7. The distribution of the value of power-law index *p* of electrons accelerated by magnetic reconnection. It is calculated by equation (5) based on the simulation data of MAD98.

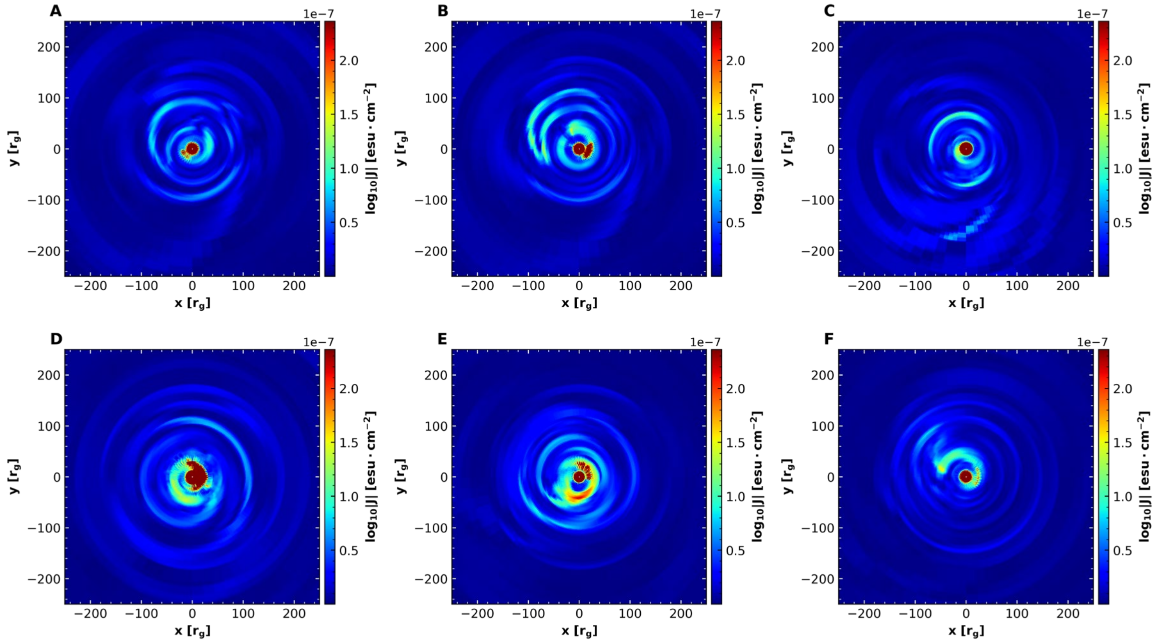

Fig. S8. The distribution of the magnitude of current density in MAD98 at different simulation times in the $x - y$ plane at $z = 600r_g$. The corresponding times of panels (**A**) - (**F**) are t=25300, 25500, 26700, 27400, 33800, and 37300 r_g/c, respectively.

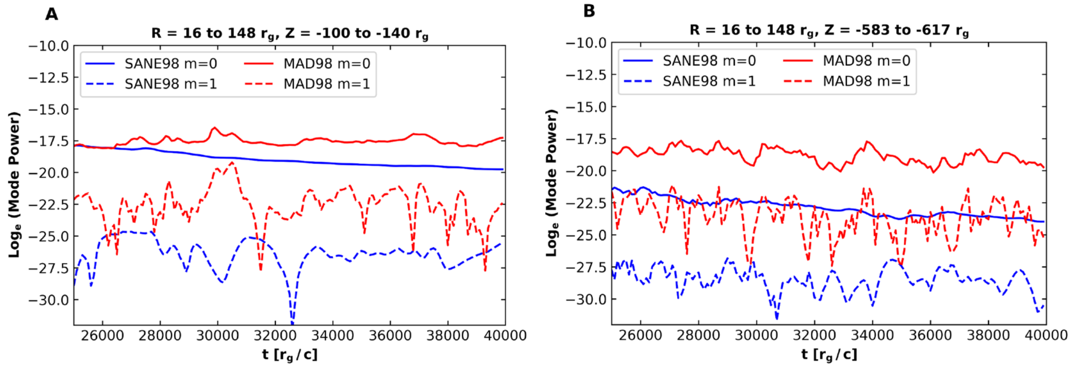

Fig. S9. The time evolution of the azimuthal Fourier power in $m = 0$ and $m = 1$ modes for SANE98 (blue) and MAD98 (red). From left to right correspond to two different regions of the jet. (**A**) At small z ~ $120r_g$. (**B**) At large z ~ $600r_g$. The larger power in MAD98 compared to SANE98 and the presence of notable $m = 1$ mode power even at small z ($\sim 120r_g$), among others, indicate that magnetic eruption rather than kink instability is likely the physical mechanism of driving reconnection.

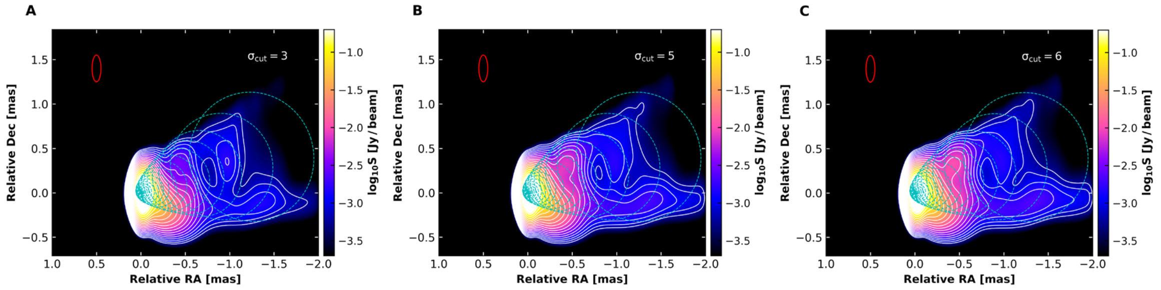

Fig. S10. The predicted images at 86 GHz by MAD98 using different values of magnetization model parameter. (**A**) $\sigma_{cut} = 3$. (**B**) $\sigma_{cut} = 5$. (**C**) $\sigma_{cut} = 6$. The three results are very similar, indicating that the modeling result is not sensitive to the choice of σ_{cut}. The dotted circles denote the boundary of the BZ-jet.

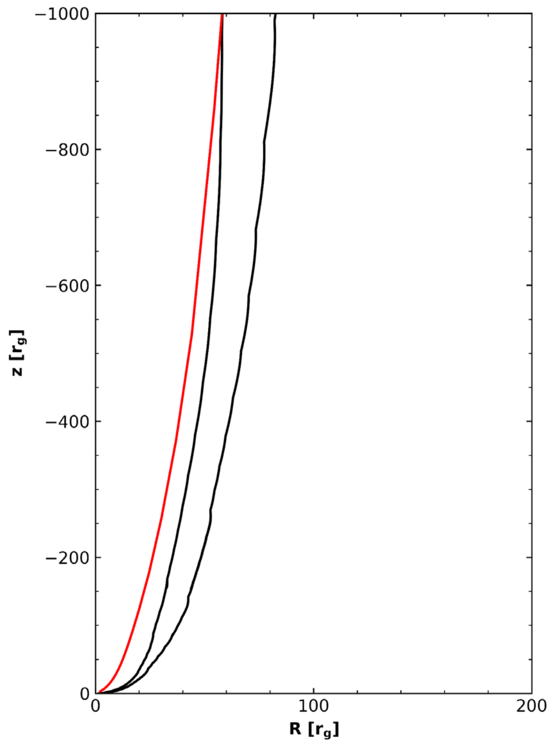

Fig. S11. The comparison between the $\sigma_{cut} = cont.$ line and streamline based on ϕ-averaged simulation data of MAD98. The red line denotes the streamline, while the two black lines denote the $\sigma_{cut} = 5$ (closer to the jet axis) and $\sigma_{cut} = 1$ (away from the jet axis) lines.

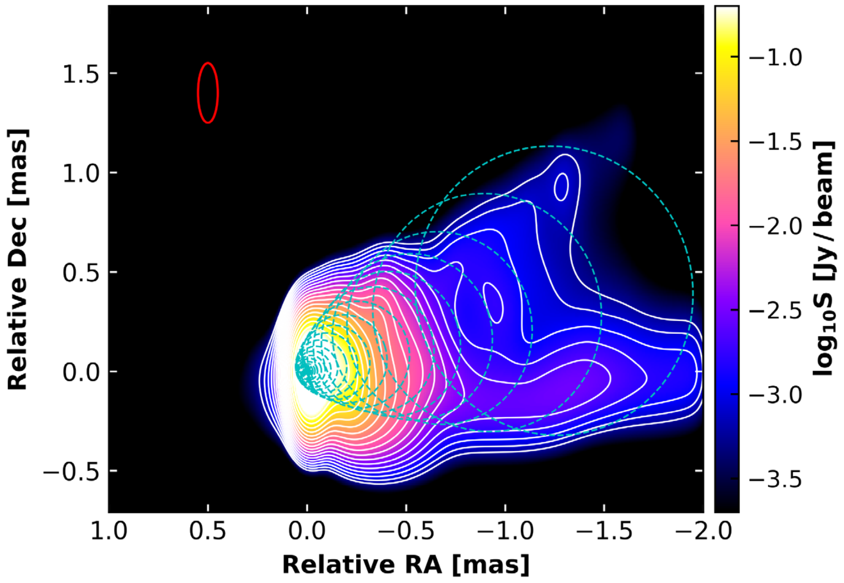

Fig. S12. The predicted image at 86 GHz using the streamline as the boundary of the radiation region in the jet. The streamline is the one shown in Fig. S11. This image is very similar to the image produced by the fiducial model shown by the top-middle panel of Fig. 1. The dotted circles denote the boundary of the BZ-jet.

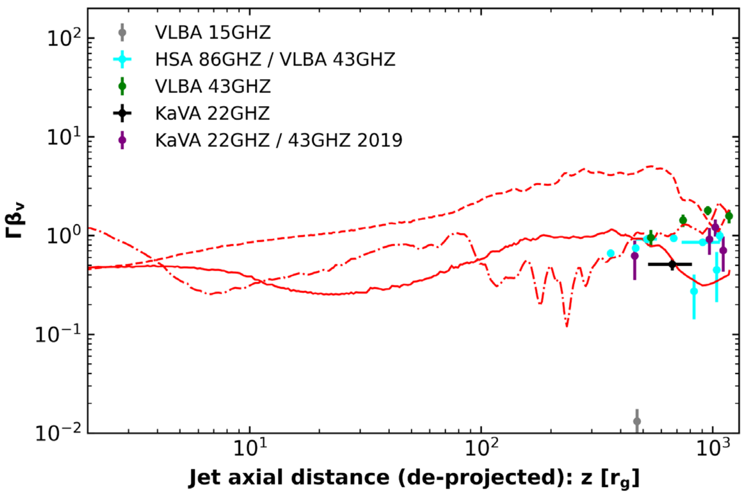

Fig. S13. Comparison of the predicted (lines) and observed (dots) jet velocity as a function of de-projected distance from the core. The dotted, dashed, and solid lines represent the distribution of $\Gamma\beta$ along the line that is located at 10%, 50%, and 100% of the outer boundary of the BZ-jet, respectively. The observational data are taken from Refs. (*67 & 68*) (VLBA 15 GHz), (*69*) (HSA 86GHZ/VLBA 43GHZ), (*70)* (KaVA 22GHz), (*71*) (VLBA 43GHz), and (72) (KaVA 22GHz/43GHz).

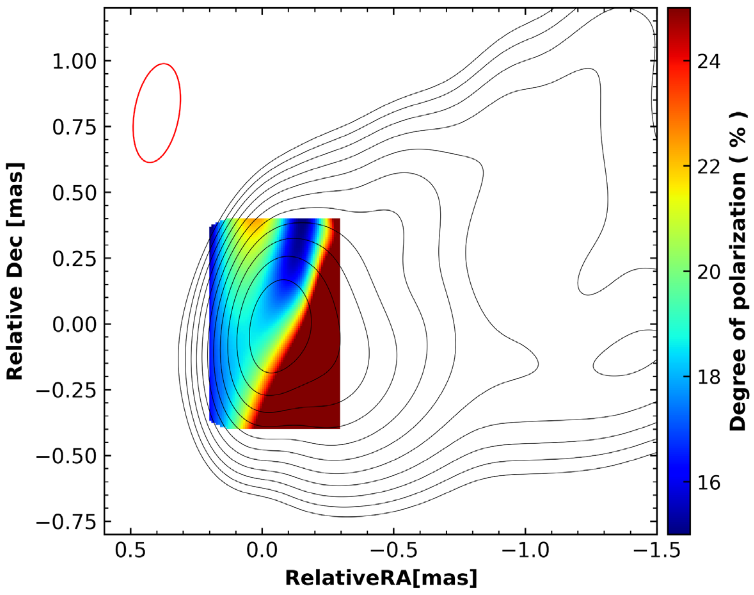

Fig. S14. The polarization degree in the nuclear region of M87 predicted by the fiducial model. For comparison with the observational result shown in Ref. (*73*), only the result in a central region is shown. Note that the predicted polarization degree shown here should be regarded as an upper limit when compared to the observational result.

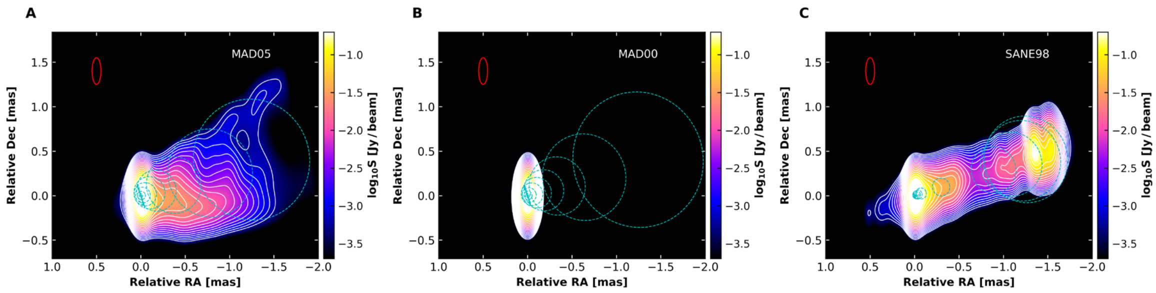

Fig. S15. The jet images predicted by different models. (**A**) MAD05, (**B**) MAD00, and (**C**) SANE98. The dotted circles denote the boundary of the BZ-jet.

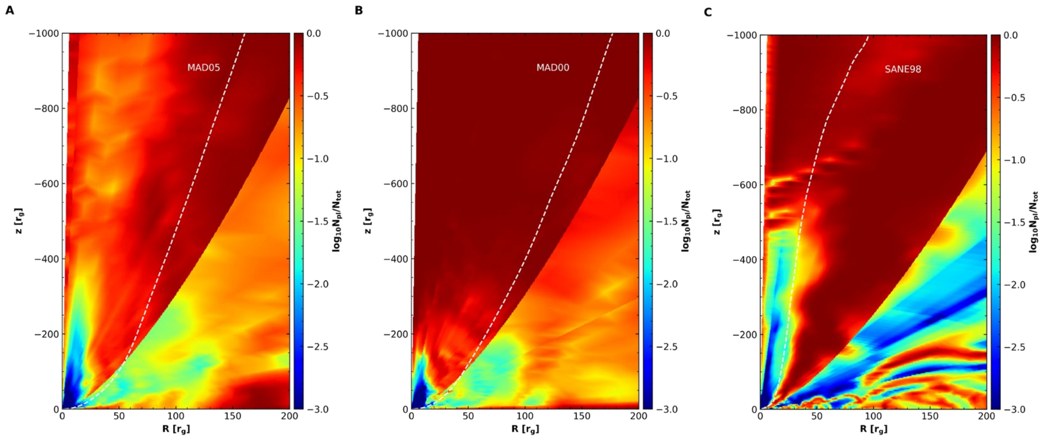

Fig. S16. The ϕ-averaged distribution of the ratio of nonthermal and total electrons number density predicted by different models. They are (**A**) MAD05, (**B**) MAD00, and (**C**) SANE98, corresponding to Fig. S15. The white dashed line in all panels denotes the ϕ-averaged boundary of the BZ-jet.

Table S1.

The static mesh refinement grid of MAD98

Level	$r\,/\,r_g$	$\theta\,/\,\pi$	$\varphi\,/\,\pi$
0	[1.1, 1200]	[0, 1]	[0, 2]
1	[1.1, 200]	[0.1305, 0.8689]	[0, 2]
2	[1.1, 30]	[0, 1]	[0, 2]
	[30, 1200]	[0.0722, 0.1667]	[0, 2]
	[30, 1200]	[0.8333, 0.9278]	[0, 2]
3	[30, 1200]	[0.0555, 0.0722]	[0, 2]
	[30, 1200]	[0.9278, 0.9444]	[0, 2]
	[30, 1200]	[0.0055, 0.0555]	[0, 2]
4	[30, 1200]	[0.9444, 0.9944]	[0, 2]

Table S2.

The static mesh refinement grid of SANE98, MAD05, and MAD00

Level	r / r_g	θ / π	φ / π
0	[1, 1200]	[0, 1]	[0, 2]
1	[1, 30]	[0, 0.1942]	[0, 2]
	[1, 30]	[0.8058, 1]	[0, 2]
	[1, 200]	[0.1305, 0.8695]	[0, 2]
2	[1, 30]	[0.1942, 0.8058]	[0, 2]
	[6.4, 1200]	[0, 0.1209]	[0, 2]
	[6.4, 1200]	[0.8791, 1]	[0, 2]
3	[36.7, 1200]	[0, 0.0573]	[0, 2]
	[36.7, 1200]	[0.9427, 1]	[0, 2]

Table S3.

The mass accretion rates of various models

Model	η	$\dot{M}$ $(10^{-3}\, M_{\odot}/yr)$
``Thermal-only'' MAD98	—	*0.103*
``Current density'' MAD98	0.2×10^{-5}	0.094
``Current density'' MAD05	1.3×10^{-5}	0.398
``Current density'' MAD00	0.47×10^{-5}	0.125
``Current density'' SANE98	0.008×10^{-5}	0.374

REFERENCE AND NOTES

1. R. D. Blandford, R. L. Znajek, Electromagnetic extraction of energy from Kerr black holes. *Mon. Not. R. Astron. Soc.* **179,** 433–456 (1977).

2. J. F. Hawley, S. A. Balbus, The dynamical structure of nonradiative black hole accretion flows. *Astrophys. J.* **573**, 738–748 (2002).

3. A. Tchekhovskoy, R. Narayan, J. C. McKinney, Efficient generation of jets from magnetically arrested accretion on a rapidly spinning black hole. *Mon. Not. R. Astron. Soc.*, **418**, L79-L83 (2011).

4. J. C. McKinney, A. Tchekhovskoy, R. D. Blandford, General relativistic magnetohydrodynamic simulations of magnetically choked accretion flows around black holes. *Mon. Not. R. Astron. Soc.,* **423**, 3083–3117 (2012)

5. A. Sadowski, R. Narayan, R. Penna, Y. Zhu, Energy, momentum and mass outflows and feedback from thick accretion discs around rotating black holes. *Mon. Not. R. Astron. Soc.*, **436**, 3856–3874 (2013).

6. R. D. Blandford, D. G. Payne, Hydromagnetic flows from accretion discs and the production of radio jets. *Mon. Not. R. Astron. Soc.* **199,** 883–903 (1982)

7. D. Lynden-Bell, On why discs generate magnetic towers and collimate jets. *Mon. Not. R. Astron. Soc.*, **341**, 1360–1372 (2003).

8. Y. Kato, S. Mineshige, K. Shibata, Magnetohydrodynamic accretion flows: Formation of magnetic tower jet and subsequent quasi-steady state. *Astrophys. J.* **605**, 307–320 (2004).

9. J. F. Hawley, J. H. Krolik, Magnetically driven jets in the kerr metric. *Astrophys. J.* **641**, 103 (2006), 116.

10. F. Yuan, Z. Gan, R. Narayan, A. Sadowski, D. Bu, X-N. Bai, Numerical Simulation of Hot Accretion Flows. III. Revisiting Wind Properties Using the Trajectory Approach. *Astrophys. J.* **804,** 101 (2015).

11. Materials and methods are available at the end of the main text while supplementary materials are available at the Science Advances website.

12. Event Horizon Telescope Collaboration, K. Akiyama, J. C. Algaba, A. Alberdi, W. Alef, R. Anantua, K. Asada, R. Azulay, A-K. Baczko, D. Ball, M. Baloković, J. Barrett, B. A. Benson, D. Bintley, L. Blackburn, R. Blundell, W. Boland, K. L. Bouman, G. C. Bower, H. Boyce, M. Bremer, C. D. Brinkerink, R. Brissenden, S. Britzen, A. E. Broderick, D. Broguiere, T. Bronzwaer, D-Y. Byun, J. E. Carlstrom, A. Chael, C-K. Chan, S. Chatterjee, K. Chatterjee, M-T. Chen, Y. Chen, P. M. Chesler, I. Cho, P. Christian, J. E. Conway, J. M. Cordes, T. M. Crawford, G. B. Crew, A. Cruz-Osorio, Y. Cui, J. Davelaar, M. De Laurentis, R. Deane, J. Dempsey, G. Desvignes, J. Dexter, S. S. Doeleman, R. P. Eatough, H. Falcke, J. Farah, V. L. Fish, E. Fomalont, H. A. Ford, R. Fraga-Encinas, P. Friberg, C. M. Fromm, A. Fuentes, P. Galison, C. F. Gammie, R. García, Z. Gelles, O. Gentaz, B. Georgiev, C. Goddi, R. Gold, J. L. Gómez, A. I. Gómez-Ruiz, M. Gu, M. Gurwell, K. Hada, D. Haggard, M. H. Hecht, R. Hesper, E. Himwich, L. C. Ho, P. Ho, M. Honma, C-W. L. Huang, L. Huang, D. H. Hughes, S. Ikeda, M. Inoue, S. Issaoun, D. J. James, B. T. Jannuzi, M. Janssen, B. Jeter, W. Jiang, A. Jimenez-Rosales, M. D. Johnson, S. Jorstad, T. Jung, M. Karami, R. Karuppusamy, T. Kawashima, G. K. Keating, M. Kettenis, D-J. Kim, J-Y. Kim, J. Kim, J. Kim, M. Kino, J. Y. Koay, Y. Kofuji, P. M. Koch, S. Koyama, M. Kramer, C. Kramer, T. P. Krichbaum, C-Y. Kuo, T. R. Lauer, S-S. Lee, A. Levis, Y-R. Li, Z. Li, M. Lindqvist, R. Lico, G. Lindahl, J. Liu, K. Liu, E. Liuzzo, W-P. Lo, A. P. Lobanov, L. Loinard, C. Lonsdale, R-S. Lu, N. R. MacDonald, J. Mao, N. Marchili, S. Markoff, D. P. Marrone, A. P. Marscher, I. Martí-Vidal, S. Matsushita, L. D. Matthews, L. Medeiros, K. M. Menten, I. Mizuno, Y. Mizuno, J. M. Moran, K. Moriyama, M. Moscibrodzka, C. Müller, G. Musoke, M. Mus, Alejandro, D. Michalik, A. Nadolski, H. Nagai, N. M. Nagar, M. Nakamura, R. Narayan, G. Narayanan, I. Natarajan, A. Nathanail, J. Neilsen, R. Neri, C. Ni, A. Noutsos, M. Nowak, H. Okino, H. Olivares, G. N. Ortiz-León, T. Oyama, F. Özel, D. C. M. Palumbo, J. Park, N. Patel, U-L. Pen, D. W. Pesce, V. Piétu, R. Plambeck, A. PopStefanija, O. Porth, F. M. Pötzl, B. Prather, J. A. Preciado-López, D. Psaltis, H.-Y. Pu, V. Ramakrishnan, R. Rao, M. G. Rawlings, A. W. Raymond, L. Rezzolla, A. Ricarte, B. Ripperda, F. Roelofs, A. Rogers, E. Ros, M. Rose, A. Roshanineshat, H. Rottmann, A. L. Roy, C. Ruszczyk, K. L. J. Rygl, S. Sánchez, D. Sánchez-Arguelles, M. Sasada, T. Savolainen, F. P. Schloerb, K-F. Schuster, L. Shao, Z. Shen, D. Small, B. W. Sohn, J. SooHoo, H. Sun, F. Tazaki, A. J. Tetarenko, P. Tiede, R. P. J. Tilanus, M. Titus, K. Toma, P. Torne, T. Trent, E. Traianou, S. Trippe, I. van Bemmel, H. J. van Langevelde, D. R. van Rossum, J. Wagner, D. Ward-Thompson, J. Wardle, J. Weintroub, N. Wex, R.

Wharton, M. Wielgus, G. N. Wong, Q. Wu, D. Yoon, A. Young, K. Young, Z. Younsi, F. Yuan, Y-F. Yuan, J. A. Zensus, G-Y. Zhao, S-S. Zhao, First M87 Event Horizon Telescope Results. VIII. Magnetic Field Structure near The Event Horizon. *Astrophys. J.* **910,** L13 (2021).

13. F. Yuan, H. Wang, H. Yang, The accretion flow in M87 is really MAD. *Astrophys. J.* **924**, 124 (2022).

14. M. Mościbrodzka, H. Falcke, H. Shiokawa, General relativistic magnetohydrodynamical simulations of the jet in M 87. *Astron. Astrophys.* **586**, A38 (2016).

15. I.V. Igumenshchev, Magnetically arrested disks and the origin of poynting jets: A numerical study. *Astrphys. J.* **677**, 317–326 (2008).

16. J. Dexter, A. Tchekhovskoy, A. Jiménez-Rosales, S.M. Ressler, M. Bauböck, Y. Dallilar, P. T. de Zeeuw, F. Eisenhauer, S. von Fellenberg, F. Gao, R. Genzel, S. Gillessen, M. Habibi, T. Ott, J. Stadler, O. Straub, F. Widmann, Sgr A* near-infrared flares from reconnection events in a magnetically arrested disc. *Mon. Not. R. Astron. Soc.* **497**, 4999–5007 (2020)

17. O. Porth, Y. Mizuno, Z. Younsi, C.M. Fromm, Flares in the Galactic Centre - I. Orbiting flux tubes in magnetically arrested black hole accretion discs. *Mon. Not. R. Astron. Soc.* **502**, 2023–2032 (2021).

18. B. Ripperda, M. Liska, K. Chatterjee, G. Musoke, A. A. Philippov, S. B. Markoff, A. Tchekhovskoy, Z. Younsi, Black hole flares: Ejection of accreted magnetic flux through 3D plasmoid-mediated reconnection. *Astrophys. J.* **924**, L32 (2022).

19. K. Chatterjee, R. Narayan, Flux eruption events drive angular momentum transport in magnetically arrested accretion flows. *Astrophys. J.* **941**, 30 (2022).

20. F. Guo, H. Li, W. Daughton, Y.-H. Liu, Formation of hard power laws in the energetic particle spectra resulting from relativistic magnetic reconnection. *Phys. Rev. Lett.*, **113**, 155005 (2014).

21. L. Sironi, A. Spitkovsky, Relativistic reconnection: An efficient source of non-thermal particles. *Astrophys. J.* **783**, L21 (2014).

22. D. Ball, L. Sironi, F. Özel, Electron and proton acceleration in trans-relativistic magnetic reconnection: Dependence on plasma beta and magnetization. *Astrophys. J.* **862**, 80 (2018)

23. G. R. Werner, D. A. Uzdensky, M. C. Begelman, B. Cerutti, K. Nalewajko, Non-thermal particle acceleration in collisionless relativistic electron-proton reconnection. *Mon. Not. R. Astron. Soc.* **473,** 4840–4861 (2018).

24. X. Li, F. Guo, Y.-H. Liu, H. Li, A model for nonthermal particle acceleration in relativistic magnetic reconnection. *Astrophys. J.* **954**, L37 (2023).

25. E. Petersen, C. Gammie, Non-thermal models for infrared flares from Sgr A*. *Mon. Not. R. Astron. Soc.* **494,** 5923–5935 (2020).

26. A. Cruz-Osorio, C. Fromm, Y. Mizuno, A. Nathanail, Z. Younsi, O. Porth, J. Davelaar, H. Falcke, M. Kramer, L. Rezzolla, State-of-the-art energetic and morphological modelling of the launching site of the M87 jet. *Nat. Astron.* **6**, 103–108 (2022).

27. M. Mościbrodzka, C. F. Gammie, IPOLE - semi-analytic scheme for relativistic polarized radiative transport. *Mon. Not. R. Astron. Soc.,* **475**, 43–54 (2018).

28. A. Chael, R. Narayan, M. D. Johnson, Two-temperature, magnetically arrested disc simulations of the jet from the supermassive black hole in M87. *Mon. Not. R. Astron. Soc.,* **486**, 2873–2895 (2019).

29. C. J. White, J. M. Stone, C. F. Gammie, An Extension of the Athena++ Code Framework for GRMHD Based on Advanced Riemann Solvers and Staggered-mesh Constrained Transport. *Astrophys. J., Suppl. Ser.* **225,** 22 (2016).

30. J. M. Stone, K. Tomida, C. J. White, K. G. Felker, The Athena++ Adaptive Mesh Refinement Framework: Design and Magnetohydrodynamic Solvers. *Astrophys. J., Suppl. Ser.* **249,** 4 (2020).

31. B. Einfeldt, On Godunov-Type Methods for Gas Dynamics. *SIAM J. Numer. Anal.* **25,** 294–318 (1988).

32. O. Porth, K. Chatterjee, R. Narayan, C. F. Gammie, Y. Mizuno, P. Anninos, J. G. Baker, M. Bugli, C. Chan, J. Davelaar, L. Del Zanna, Z. B. Etienne, P. C. Fragile, B. J. Kelly, M. Liska, S. Markoff, J. C.

McKinney, B. Mishra, S. C. Noble, H. Olivares, B. Prather, L. Rezzolla, B. R. Ryan, J. M. Stone, N. Tomei, C. J. White, Z. Younsi, K. Akiyama, A. Alberdi, W. Alef, K. Asada, R. Azulay, A.-K. Baczko, D. Ball, M. Baloković, J. Barrett, D. Bintley, L. Blackburn, W. Boland, K. L. Bouman, G. C. Bower, M. Bremer, C. D. Brinkerink, R. Brissenden, S. Britzen, A. E. Broderick, D. Broguiere, T. Bronzwaer, D.-Y. Byun, J. E. Carlstrom, A. Chael, S. Chatterjee, M.-T. Chen, Y. Chen, I. Cho, P. Christian, J. E. Conway, J. M. Cordes, Geoffrey, B. Crew, Y. Cui, M. De Laurentis, R. Deane, J. Dempsey, G. Desvignes, S. S. Doeleman, R. P. Eatough, H. Falcke, V. L. Fish, E. Fomalont, R. Fraga-Encinas, B. Freeman, P. Friberg, C. M. Fromm, J. L. Gómez, P. Galison, R. García, O. Gentaz, B. Georgiev, C. Goddi, R. Gold, M. Gu, M. Gurwell, K. Hada, M. H. Hecht, R. Hesper, L. C. Ho, P. Ho, M. Honma, C.-W. Huang, L. Huang, D. H. Hughes, S. Ikeda, M. Inoue, S. Issaoun, D. J. James, B. T. Jannuzi, M. Janssen, B. Jeter, W. Jiang, M. D. Johnson, S. Jorstad, T. Jung, M. Karami, R. Karuppusamy, T. Kawashima, G. K. Keating, M. Kettenis, J.-Y. Kim, J. Kim, J. Kim, M. Kino, J. Y. Koay, Patrick, M. Koch, S. Koyama, M. Kramer, C. Kramer, T. P. Krichbaum, C.-Y. Kuo, T. R. Lauer, S.-S. Lee, Y.-R. Li, Z. Li, M. Lindqvist, K. Liu, E. Liuzzo, W.-P. Lo, A. P. Lobanov, L. Loinard, C. Lonsdale, R.-S. Lu, N. R. MacDonald, J. Mao, D. P. Marrone, A. P. Marscher, I. Martí-Vidal, S. Matsushita, L. D. Matthews, L. Medeiros, K. M. Menten, I. Mizuno, J. M. Moran, K. Moriyama, M. Moscibrodzka, C. Müller, H. Nagai, N. M. Nagar, M. Nakamura, G. Narayanan, I. Natarajan, R. Neri, C. Ni, A. Noutsos, H. Okino, T. Oyama, F. Özel, D. C. M. Palumbo, N. Patel, U.-L. Pen, D. W. Pesce, V. Piétu, R. Plambeck, A. PopStefanija, J. A. Preciado-López, D. Psaltis, H.-Y. Pu, V. Ramakrishnan, R. Rao, M. G. Rawlings, A. W. Raymond, B. Ripperda, F. Roelofs, A. Rogers, E. Ros, M. Rose, A. Roshanineshat, H. Rottmann, A. L. Roy, C. Ruszczyk, K. L. J. Rygl, S. Sánchez, D. Sánchez-Arguelles, M. Sasada, T. Savolainen, F. P. Schloerb, K.-F. Schuster, L. Shao, Z. Shen, D. Small, B. W. Sohn, J. SooHoo, F. Tazaki, P. Tiede, R. P. J. Tilanus, M. Titus, K. Toma, P. Torne, T. Trent, S. Trippe, S. Tsuda, I. van Bemmel, H. J. van Langevelde, D. R. van Rossum, J. Wagner, J. Wardle, J. Weintroub, N. Wex, R. Wharton, M. Wielgus, G. N. Wong, Q. Wu, K. Young, A. Young, F. Yuan, Y.-F. Yuan, J. A. Zensus, G. Zhao, S.-S. Zhao, Z. Zhu, Event Horizon Telescope Collaboration, The event horizon general relativistic magnetohydrodynamic code comparison project. *Astrophys. J. Supp.* **243,** 26 (2019).

33. J. C. McKinney, C. F. Gammie, A Measurement of the electromagnetic luminosity of a Kerr black Hole. *Astrophys. J.* **611,** 977–995 (2004).

34. R. Narayan, I. V. Igumenshchev, M. A. Abramowicz, Magnetically arrested disk: An energetically efficient accretion flow. *Publ. Astron. Soc. Jpn.* **55,** L69–L72 (2003).

35. R. Narayan, A. SÄdowski, R. F. Penna, A. K. Kulkarni, GRMHD simulations of magnetized advection-dominated accretion on a non-spinning black hole: Role of outflows. *Mon. Not. R. Astron. Soc.* **426,** 3241–3259 (2012)

36. L. G. Fishbone, V. Moncrief, Relativistic fluid disks in orbit around Kerr black holes. *Astrophys. J.* **207,** 962–976 (1976).

37. R. F. Penna, A. Kulkarni, R. Narayan, A new equilibrium torus solution and GRMHD initial conditions. *Astron. Astrophys.* **559,** A116 (2013).

38. C. J. White, F. Chrystal, The effects of resolution on black hole accretion simulations of jets. *Mon. Not. R. Astron. Soc.* **498,** 2428–2439 (2020).

39. J. F. Hawley, X. Guan, J. H. Krolik, Assessing quantitative results in accretion simulations: From local to global. *Astrophys. J.* **738**, 84 (2011).

40. R.-S. Lu, K. Asada, T. P. Krichbaum, J. Park, F. Tazaki, H.-Y. Pu, M. Nakamura, A. Lobanov, K. Hada, K. Akiyama, J-Y. Kim, I. Marti-Vidal, J. L. Gomez, T. Kawashima, F. Yuan, E. Rose, W. Alef, S. Britzen, M. Bremer, P. T. P. Ho, M. Honma, D. H. Hughes, M. Inoue, W. Jiang, M. Kino, S. Koyama, M. Lindqvist, J. Liu, A. P. Marscher, S. Matsushita, H. Nagai, H. Rottmann, T. Savolainen, K-F. Schuster, Z-Q. Shen, P. de Vicente, R. C. Walker, H. Yang, J. A. Zensus, J. C. Algaba, A. Allardi, U. Bach, R. Berthold, D. Bintley, D-Y. Byun, C. Casadio, S-H. Chang, C-C. Chang, S-C. Chang, C-C. Chen, M-T. Chen, R. Chilson, T. C. Chuter, J. Conway, G. B. Crew, J. T. Dempsey, S. Dornbusch, A. Faber, P. Friberg, J. G. Garcia, M. G. Garrido, C-C. Han, K.-C. Han,Y. Hasegawa, R. Herrero-Illana, Y.-D. Huang, C.-W. L. Huang, V. Impellizzeri, H. Jiang, H. Jinchi, T. Jung, J. Kallunki, P. Kirves, K. Kimura, J. Y. Koay, P. M. Koch, C. Kramer, A. Kraus, D. Kubo, C.-Y. Kuo, C.-T. Li, L. C.-C. Lin, C.-T. Liu, K.-Y. Liu, W.-P. Lo, L.-M. Lu, N. MacDonald, P. Martin-Cocher, H. Messias, Z. Meyer-Zhao, A. Minter, D. G. Nair, H. Nishioka, T. J. Norton, G. Nystrom, H. Ogawa, P. Oshiro, N. A. Patel, U.-L. Pen, Y. Pidopryhora, N. Pradel, P. A. Raffin, R. Rao, I. Ruiz, S. Sanchez, P. Shaw, W. Snow, T. K. Sridharan, R. Srinivasan, B. Tercero, P. Torne, E. Traianou, J. Wagner, C. Walther, T-S. Wei, J. Yang,

C.-Y. Yu, A ring-like accretion structure in M87 connecting its black hole and jet. *Nature.* **616**, 686–690 (2023).

41. Q. Zhang, F. Guo, W. Daughton, H. Li, X. Li, Efficient nonthermal ion and electron acceleration enabled by the flux-rope kink instability in 3D nonrelativistic magnetic reconnection. *Phys. Rev. Lett.*, **127**, 185101 (2021).

42. F. Özel, D. Psaltis, R. Narayan, Hybrid thermal-nonthermal synchrotron emission from hot accretion flows. *Astrophys. J.* **541,** 234 (2000), 249.

43. F. Yuan, E. Quataert, R. Narayan, Nonthermal electrons in radiatively inefficient accretion flow models of Sagittarius A*. *Astrophys. J.* **598,** 301 (2003), 312.

44. J. Dexter, J. C. McKinney, E. Agol, The size of the jet launching region in M87. *Mon. Not. R. Astron. Soc.* **421,** 1517 (2012), 1528.

45. K. Chatterjee, S. Markoff, J. Neilsen, Z. Younsi, G. Witzel, A. Tchekhovskoy, D. Yoon, A. Ingram, M. van der Klis, H. Boyce, T. Do, D. Haggard, M. A. Nowak, General relativistic MHD simulations of non-thermal flaring in Sagittarius A*. *Mon. Not. R. Astron. Soc.,* **507**, 5281–5302 (2021).

46. A. E. Broderick, V. L. Fish, M. D. Johnson, K. Rosenfeld, C. Wang, S. S. Doeleman, K. Akiyama, T. Johannsen, A. L. Roy, Modeling seven years of event horizon telescope observations with radiatively inefficient accretion flow models. *Astrophys. J.* **820,** 137 (2016).

47. J. Davelaar, M. Mościbrodzka, T. Bronzwaer, H. Falcke, General relativistic magnetohydrodynamicalκ-jet models for Sagittarius A*. *Astron. Astrophys.* **612,** A34 (2018).

48. A. E. Broderick, J. C. McKinney, Parsec-scale faraday rotation measures from general relativistic magnetohydrodynamic simulations of active galactic nucleus jets. *Astrophys. J.* **725,** 773 (2010).

49. J. Davelaar, H. Olivares, O. Porth, T. Bronzwaer, M. Janssen, F. Roelofs, Y. Mizuno, C. M. Fromm, H. Falcke, L. Rezzolla, Modeling non-thermal emission from the jet-launching region of M 87 with adaptive mesh refinement. *Astron. Astrophys.* **632,** A2 (2019).

50. D. Ball, F. Özel, D. Psaltis, C. K. Chan, L. Sironi, The properties of reconnection current sheets in GRMHD simulations of radiatively inefficient accretion flows, *Astrophys. J.* **853**, 184 (2018).

51. C. B. Singh, Y. Mizuno, E. M. de Gouveia Dal Pino, Spatial growth of current-driven instability in relativistic rotating jets and the search for magnetic reconnection. *Astrophys. J.* **824**, 48 (2016).

52. E. P. Alves, J. Zrake, F. Fiuza, efficient nonthermal particle acceleration by the kink instability in relativistic jets. *Phys. Rev. Lett.*, **121**, 245101 (2018)

53. T. E. Medina-Torrejón, E. M. de Gouveria Dal Pino, L. H. S. Kadowaki, G. Kowal, C. B. Singh, Y. Mizuno, Particle acceleration by relativistic magnetic reconnection driven by kink instability turbulence in poynting flux-dominated jets. *Astrophys. J.* **908**, 193 (2021).

54. A. Tomimatsu, T. Matsuoka, M. Takahashi, Screw instability in black hole magnetospheres and a stabilizing effect of field-line rotation. *Phys.Rev. D.* **64**, 123003 (2001).

55. M. Nakamura, H. Li, S. T. Li, Stability properties of magnetic tower jets. *Astrophys. J.* **656,** 721–732 (2007).

56. H. Yang, F. Yuan, Y. F. Yuan, C. White, Numerical simulation of hot accretion flows (IV): Effects of black hole spin and magnetic field strength on the wind and the comparison between wind and jet properties. *Astrophys. J.* **914,** 131 (2021).

57. K. Nishikawa, Y. Mizuno, J. L. Gomez, I. Dutan, J. Niemiec, O. Kobzar, N. MacDonald, A. Meli, M. Pohl, K. Hirotani, Rapid particle acceleration due to recollimation shocks and turbulent magnetic fields in injected jets with helical magnetic fields. *Mon. Not. R. Astron. Soc.* **493,** 2652–2658 (2020).

58. L. Sironi, M. E. Rowan, R. Narayan, Reconnection-driven particle acceleration in relativistic shear flows. *Astrophys. J.* **907**, L44 (2021).

59. B. R. Ryan, S. M. Ressler, J. C. Dolence, C. Gammie, E. Quataert, Two-temperature GRRMHD simulations of M87. *Astrophys. J.* **864,** 126 (2018).

60. Event Horizon Telescope Collaboration, K. Akiyama, A. Alberdi, W. Alef, K. Asada, R. Azulay, A.-K. Baczko, D. Ball, M. Baloković, J. Barrett, D. Bintley, L. Blackburn, W. Boland, K. Bouman, G. C.

Bower, M. Bremer, C. D. Brinkerink, R. Brissenden, S. Britzen, A. E. Broderick, D. Broguiere, T. Bronzwaer, D.-Y. Byun, J. E. Carlstrom, A. Chael, C. Chan, S. Chatterjee, K. Chatterjee, M.-T. Chen, Y. Chen, I. Cho, P. Christian, J. E. Conway, J. M. Cordes, G. B. Crew, Y. Cui, J. Davelaar, M. De Laurentis, R. Deane, J. Dempsey, G. Desvignes, J. Dexter, S. S. Doeleman, R. P. Eatough, H. Falcke, V. L. Fish, E. Fomalont, R. Fraga-Encinas, P. Friberg, C. M. Fromm, J. L. Gómez, P. Galison, C. F. Gammie, R. García, O. Gentaz, B. Georgiev, C. Goddi, R. Gold, M. Gu, M. Gurwell, K. Hada, M. H. Hecht, R. Hesper, L. C. Ho, P. Ho, M. Honma, C.-W. L. Huang, L. Huang, D. H. Hughes, S. Ikeda, M. Inoue, S. Issaoun, D. J. James, B. T. Jannuzi, M. Janssen, B. Jeter, W. Jiang, M. D. Johnson, S. Jorstad, T. Jung, M. Karami, R. Karuppusamy, T. Kawashima, G. K. Keating, M. Kettenis, J.-Y. Kim, J. Kim, J. Kim, M. Kino, J. Y. Koay, P. M. Koch, S. Koyama, M. Kramer, C. Kramer, T. P. Krichbaum, C.-Y. Kuo, T. R. Lauer, S.-S. Lee, Y.-R. Li, Z. Li, M. Lindqvist, K. Liu, E. Liuzzo, W.-P. Lo, A. P. Lobanov, L. Loinard, C. Lonsdale, R.-S. Lu, N. R. MacDonald, J. Mao, S. Markoff, D. P. Marrone, A. P. Marscher, I. Martí-Vidal, S. Matsushita, L. D. Matthews, L. Medeiros, K. M. Menten, Y. Mizuno, I. Mizuno, J. M. Moran, K. Moriyama, M. Moscibrodzka, C. MuÏler, H. Nagai, N. M. Nagar, M. Nakamura, R. Narayan, G. Narayanan, I. Natarajan, R. Neri, C. Ni, A. Noutsos, H. Okino, H. Olivares, T. Oyama, F. Özel, D. C. M. Palumbo, N. Patel, U.-L. Pen, D. W. Pesce, V. Piétu, R. Plambeck, A. PopStefanija, O. Porth, B. Prather, J. A. Preciado-López, D. Psaltis, H.-Y. Pu, V. Ramakrishnan, R. Rao, M. G. Rawlings, A. W. Raymond, L. Rezzolla, B. Ripperda, F. Roelofs, A. Rogers, E. Ros, M. Rose, A. Roshanineshat, H. Rottmann, A. L. Roy, C. Ruszczyk, B. R. Ryan, K. L. J. Rygl, S. Sánchez, D. Sánchez-Arguelles, M. Sasada, T. Savolainen, F. P. Schloerb, K.-F. Schuster, L. Shao, Z. Shen, D. Small, B. W. Sohn, J. SooHoo, F. Tazaki, P. Tiede, R. P. J. Tilanus, M. Titus, K. Toma, P. Torne, T. Trent, S. Trippe, S. Tsuda, I. van Bemmel, H. J. van Langevelde, D. R. van Rossum, J. Wagner, J. Wardle, J. Weintroub, N. Wex, R. Wharton, M. Wielgus, G. N. Wong, Q. Wu, A. Young, K. Young, Z. Younsi, F. Yuan, Y.-F. Yuan, J. A. Zensus, G. Zhao, S.-S. Zhao, Z. Zhu, J. Anczarski, F. K. Baganoff, A. Eckart, J. R. Farah, D. Haggard, Z. Meyer-Zhao, D. Michalik, A. Nadolski, J. Neilsen, H. Nishioka, M. A. Nowak, N. Pradel, R. A. Primiani, K. Souccar, L. Vertatschitsch, P. Yamaguchi, S. Zhang, First M87 event horizon telescope results. V. physical origin of the asymmetric ring. *Astrophys. J.* **875**, L5 (2019).

61. K. Gebhardt, J. Adams, D. Richstone, T. R. Lauer, S. M. Faber, K. Gultekin, J. Murphy, S. Tremaine, The black hole mass in M87 from Gemini/NIFS adaptive optics observations. *Astrophys. J.* **729,** 119 (2011).

62. R. C. Walker, P. E. Hardee, F. B. Davies, C. Ly, W. Junor, The structure and dynamics of the subparsec jet in M87 based on 50 VLBA observations over 17 years at 43 GHz. *Astrophys. J.* **855,** 128 (2018).

63. K. Asada, M. Nakamura, The structure of the M87 jet: A transition from parabolic to conical streamlines. *Astrophys. J.* **745**, L28 (2012).

64. J.-Y. Kim, T. P. Krichbaum, R.-S. Lu, E. Ros, U. Bach, M. Bremer, P. de Vicente, M. Lindqvist, J. A. Zensus, The limb-brightened jet of M87 down to the 7 Schwarzschild radii scale. *Astron. Astrophys.* **616,** A188 (2018).

65. M. Janssen, C. Goddi, I. M. van Bemmel, M. Kettenis, D. Small, E. Liuzzo, K. Rygl, I. Marti-Vidal, L. Blackburn, M. Wielgus, H. Falcke, rPICARD: A CASA-based calibration pipeline for VLBI data. Calibration and imaging of 7 mm VLBA observations of the AGN jet in M 87. arXiv: 1902.01749 [astro-ph.IM] (15 May 2019).

66. M. Nakamura, K. Asada, K. Hada, H.-Y. Pu, S. Noble, C. Tseng, K. Toma, M. Kino, H. Nagai, K. Takahashi, J-C Algaba, M. Orienti, K. Akiyama, A. Doi, G. Giovannini, M. Giroletti, M. Honma, S. Koyama, R. Lico, K. Niinuma, F. Tazaki, Parabolic Jets from the Spinning Black Hole in M87. *Astrophys. J.* **868**, 146 (2018).

67. K. I. Kellermann, M. L. Lister, D. C. Homan, R. C. Vermeulen, M. H. Cohen, E. Ros, M. Kadler, J. A. Zensus, Y. Y. Kovalev, Sub-milliarcsecond imaging of quasars and active galactic nuclei. III. kinematics of parsec-scale radio jets. *Astrophys. J.* **609**, 539–563 (2004).

68. Y. Y. Kovalev, M. L. Lister, D. C. Homan, K. I. Kellermann, The inner jet of the radio galaxy M87. *Astrophys. J.* **668**, L27-L30 (2007).

69. K. Hada, M. Kino, A. Doi, H. Nagai, M. Honma, K. Akiyama, F. Tazaki, R. Lico, M. Giroletti, G. Giovannini, M. Orienti, Y. Hagiwara, High-sensitivity 86 GHz (3.5 mm) VLBI observations of M87: Deep imaging of the jet base at a resolution of 10 Schwarzschild radii. *Astrophys. J.* **817**, 131 (2016).

70. K. Hada, J. H. Park, M. Kino, K. Niinuma, B. W. Sohn, H. W. Ro, T. Jung, J.-C. Algaba, G.-Y. Zhao, S.-S. Lee, K. Akiyama, S. Trippe, K. Wajima, S. Sawada-Satoh, F. Tazaki, I. Cho, J. Hodgson, J. A. Lee, Y. Hagiwara, M. Honma, S. Koyama, J. Oh, T. Lee, H. Yoo, N. Kawaguchi, D-G. Roh, S.-J. Oh,

J.-H. Yeom, D.-K. Jung, C. Oh, H.-R. Kim, J.-Y. Hwang, D.-Y. Byun, S.-H. Cho, H.-G. Kim, H. Kobayashi, K. M. Shibata, Pilot KaVA monitoring on the M 87 jet: Confirming the inner jet structure and superluminal motions at sub-pc scales. *Publ. Astron. Soc. Jpn.*, **69**, 71 (2017).

71. F. Mertens, A. P. Lobanov, R. C. Walker, P. E. Hardee, Kinematics of the jet in M 87 on scales of 100-1000 Schwarzschild radii. *Astron. Astrophys.* **595**, A54 (2016).

72. J. Park, K. Hada, M. Kino, M. Nakamura, J. Hodgson, H. Ro, Y. Cui, K. Asada, J-C. Algaba, S. Sawada-Satoh, S-S. Lee, I. Cho, Z. Shen, W. Jiang, S. Trippe, K. Niinuma, B. W. Sohn, T. Jung, G.-Y. Zhao, K. Wajima, F. Tazaki, M. Honma, T. An, K. Akiyama, D.-Y. Byun, J. Kim, Y. Zhang, X. Cheng, H. Kobayashi, K. M. Shibata, J. W. Lee, D.-G. Roh, S.-J. Oh, J.-H. Yeom, D.-K. Jung, C. Oh, H.-R. Kim, J.-Y. Hwang, Y. Hagiwara, Kinematics of the M87 Jet in the collimation zone: Gradual acceleration and velocity stratification. *Astrophys. J.* **887**, 147 (2019).

73. E. Kravchenko, M. Giroletti, K. Hada, D. L. Meier, M. Nakamura, J. Park, R. C. Walker, Linear polarization in the nucleus of M87 at 7 mm and 1.3 cm. *Astron. Astrophys.* **637,** L6 (2020).

74. J. Park, K. Hada, M. Kino, M. Nakamura, H. Ro, and S. Trippe, Faraday rotation in the Jet of M87 inside the Bondi radius: Indication of winds from hot accretion flows confining the relativistic jet. *Astrophys. J.* **871**, 257 (2019).

75. V. S. Beskin, Ya. N. Istomin, and V. I. Parev, Filling the magnetosphere of a supermassive black hole with plasma. *Sov. Astron.* **36**, 642 (1992).

76. A. Levinson, F. Rieger, Variable TeV EMISSION as a manifestation of jet formation in M87?. *Astrophys. J.* **730**, 123 (2011).

77. A. E. Broderick, A. Tchekhovskoy, Horizon-scale lepton acceleration in jets: Explaining the compact radio emission in M87. *Astrophys. J.* **809**, 97 (2015).

78. A. A. Zdziarski, D. G. Phuravhathu, M. Sikora, M. Böttcher, J. O. Chibueze, The composition and power of the jet of the broad-line radio galaxy 3C 120. *Astrophys. J.* **928**, L9 (2022).

79. E. E. Nokhrina, V. S. Beskin, Y. Y. Kovalev, A. A. Zheltoukhov, Intrinsic physical conditions and structure of relativistic jets in active galactic nuclei. *Mon. Not. R. Astron. Soc.* **447**, 2726–2737 (2015).
